# Light distribution in fat cell layers at physiological temperatures

**DOI:** 10.1038/s41598-022-25012-9

**Published:** 2023-01-19

**Authors:** Irina Yu. Yanina, Polina A. Dyachenko, Arkady S. Abdurashitov, Alexander S. Shalin, Igor V. Minin, Oleg V. Minin, Andrey D. Bulygin, Denis A. Vrazhnov, Yury V. Kistenev, Valery V. Tuchin

**Affiliations:** 1 Science Medical Center, Saratov State University, 83 Astrakhanskaya Str., Saratov, Russia 410012; 2 Laboratory of Laser Molecular Imaging and Machine Learning, Tomsk State University, 36 Lenin’s Av., Tomsk, Russia 634050; 3 Center for Neurobiology and Brain Restoration, Skolkovo Institute of Science and Technology, 3Nobelya Str., Moscow, Russia 121205; 4 Center for Photonics and 2D Materials, Moscow Institute of Physics and Technology, Dolgoprudny, 141700 Russia; 5 Institute of Telecommunications, Riga Technical University, 12 Azenes str., LV-1658 Riga, Latvia; 6 Laboratory of Fiber Optics and Optical Measurements UB-1, Kotel’nikov Institute of Radio Engineering and Electronics of Russian Academy of Sciences (Ulyanovsk Branch), 48 Goncharova Str., Ulyanovsk, Russia 432011; 7 School of Nondestructive Testing, Tomsk Polytechnic University, 30 Lenin Av., Tomsk, Russia 634050; 8 Institute for Strategic Studies, Siberian State University of Geosystems and Technologies, 10 Plahotnogo Str., Novosibirsk, Russia 630108; 9 Laboratory of Nonlinear Optical Interactions, V.E. Zuev Institute of Atmospheric Optics of Siberian Branch of the Russian Academy of Sciences, 1 Academician Zuev Sq., Tomsk, Russia 634055; 10 Laboratory for Remote Sensing of the Environment, V.E. Zuev Institute of Atmospheric Optics of Siberian Branch of the Russian Academy of Sciences, 1 Academician Zuev Sq., Tomsk, Russia 634055; 11 Laboratory of Laser Diagnostics of Technical and Living Systems, Institute of Precision Mechanics and Control, FRC “Saratov Scientific Centre of the Russian Academy of Sciences”, 24 Rabochaya Str., Saratov, Russia 410028; 12 A.N. Bach Institute of Biochemistry, FRC “Fundamentals of Biotechnology”, 33-2, Leninsky Av., Moscow, Russia 119991

**Keywords:** Biophotonics, Computational models

## Abstract

Adipose tissue (AT) optical properties for physiological temperatures and in vivo conditions are still insufficiently studied. The AT is composed mainly of packed cells close to spherical shape. It is a possible reason that AT demonstrates a very complicated spatial structure of reflected or transmitted light. It was shown with a cellular tissue phantom, is split into a fan of narrow tracks, originating from the insertion point and representing filament-like light distribution. The development of suitable approaches for describing light propagation in a AT is urgently needed. A mathematical model of the propagation of light through the layers of fat cells is proposed. It has been shown that the sharp local focusing of optical radiation (light localized near the shadow surface of the cells) and its cleavage by coupling whispering gallery modes depends on the optical thickness of the cell layer. The optical coherence tomography numerical simulation and experimental studies results demonstrate the importance of sharp local focusing in AT for understanding its optical properties for physiological conditions and at AT heating.

## Introduction

Light propagation in inhomogeneous, disordered media is still an enigmatic problem with unpredictable output, as complex multiparticle light scattering results in uncountable phase delays from scattered photons^1,2^. Laser radiation falling on living tissue passes through a complex structure of cells and extracellular matrix that strongly influences the spatial distribution of light intensity manifested in the appearance of bright lines, caustics, and speckle patterns^1–3^. One of the mechanisms is multiple reflection of laser radiation by biological cell membranes. It was shown that laser radiation entering the free liquid soap solution bubble film^4^, serving as a cell membrane model, is split into a fan of narrow tracks, originating from the insertion point and representing filament-like light distribution in the film, i.e. light can be localized within photonic streams (jets) and bright spots (speckles). This effect was also observed in the liquid films of bioorganic substances^5^—the free liquid films of the aqueous gelatin and gelatin-in-glycerol solutions. Specific micro-optic effects were found in cyanobacteria^6^. The *Synechocystis* cells directly and accurately sense the position of a light source because each cell acts as a spherical microlens, allowing the cell to see a light source and move toward it. A high-resolution image of the light source is focused on the edge of the cell opposite to the source, triggering movement away from the focused spot. A dielectric sphere with properties similar to a cell (diameter of 3 µm and refractive index of 1.4) was shown to produce sharply focused light beams in the nearfield—so-called photonic jets, valid for both spherical and nonspherical particles^6,7^. A photonic jet is a narrow high-intensity non-evanescent light beam that can propagate over a distance longer than the wavelength λ^8^.

Each cell or tissue structure has a well-defined structural composition. Therefore, when the tissue has a few layers and light may not be able to acquire a random path, radiation transfer theory is not suitable to describe this process^3^. The effects of focusing incident light on the convexly curved walls of epidermal leaf cells are observed^9^. Such focusing should capture the photon flux and its higher density in certain places within the leaf and ensure more light utilization efficiency^10^. The specific light transport and focusing due to the different shapes of the soma and the nucleus of the neurons resulting in an intensity increase was analyzed based on the Mie scattering theory^11^. The live-cell lens effect was recently demonstrated for suspended red blood cells (RBCs) that behave as adaptive liquid microlenses with a tunable focal length^12^. Any epithelial or loose connective tissue, such as adipose tissue (AT) or lungs, is composed mainly of packed cells or alveolar structures close to spherical shape^3,13,14^.

Despite the long story of biological tissue optics, the AT optical properties for physiological temperatures and in vivo conditions are still insufficiently studied^15,16^. At the same time, this knowledge is vital for diagnostics, prophylactics, and treatment of diseases associated with human overweight and obesity, the discrimination between benign and tumor sites of the breast using the optical parameter related to the absorption of fat and water in the extended NIR region (1000–1600 nm)^17^, and reliable layer-by-layer dosimetry of laser radiation used in the treatment of obesity and cellulite^17–19^.

There is a relationship between some diseases and excess body fat, including type 2 diabetes mellitus, cardiovascular diseases, such as hypertension and atherosclerosis, and certain types of cancer^20,21^. The knowledge of relations between AT optical properties and adipocyte metabolism is vital, including the information on the dynamics of the morphology of fat cellsduring heating^22–25^. For example, Salomatina et al. demonstrate the relationship between morphological and spectral characteristics of AT^22^.

Hyperthermia is one of the ways to provide a controllable impact on fat cells^18^. Recent AT morphology and functioning studies were performed using noninvasive and high-resolution optical techniques with endogenous contrast^26–29^. Functional imaging of adipocyte metabolism with the subcellular resolution was conducted using intrinsic two-photon excited fluorescence^28^. Quantification of both the mechanical properties and chemical composition of AT simultaneously was performed using Brillouin and Raman microspectroscopies^29^. High-resolution functional imaging of white AT (WAT) and brown AT (BAT) cells in mice in vivo was conducted using multimodal nonlinear optical microscopy and fiber-based spectroscopy^26^. The label-free quantitative in vivo monitoring of the browning process in a mouse model was described using diffuse reflectance spectroscopy in the NIR-II optical window (1050 to 1350 nm) with a fiber probe^26^.

Imaging of an endogenous biomarker of oxidative stress in the lipid droplet (LD) of adipocytes was provided using a combination of fluorescence lifetime imaging with third-harmonic generation (THG) and Coherent Anti-Stokes Raman Scattering (CARS) microscopies^30^. Nonlinear optical microscopy methods, in particular, THG and two-photon excited autofluorescence, are also used to visualize the formation of LDs of adipocytes and to indicate the cell metabolic state associated with fatty acid synthesis and lipid accumulation^31^.

Adipocytes’ inherent mechanical properties in the course of adipogenesis (responsible for increasing fat tissue mass) were studied with interferometric phase microscopy^32^. LDs were found to be mechanically stiffer than the surrounding cytoplasm. It opens a way for investigating adipose-related diseases (overweight and obesity) based on optical studies of cellular mechanics. Quantification of individual LDs in live hepatocytes that could be involved in a number of diseases, such as diabetes mellitus or cancer, was analyzed by three-dimensional label-free optical imaging^33^. The latter is based on quantitative phase imaging and measures LD structural distribution, biochemical parameters, and dynamics. Confocal microscopy was applied for subcellular spatial resolution optical adipocyte sectioning in human dermal WAT, allowing tissue spatial reconstruction^34^.

**Figure 1:**
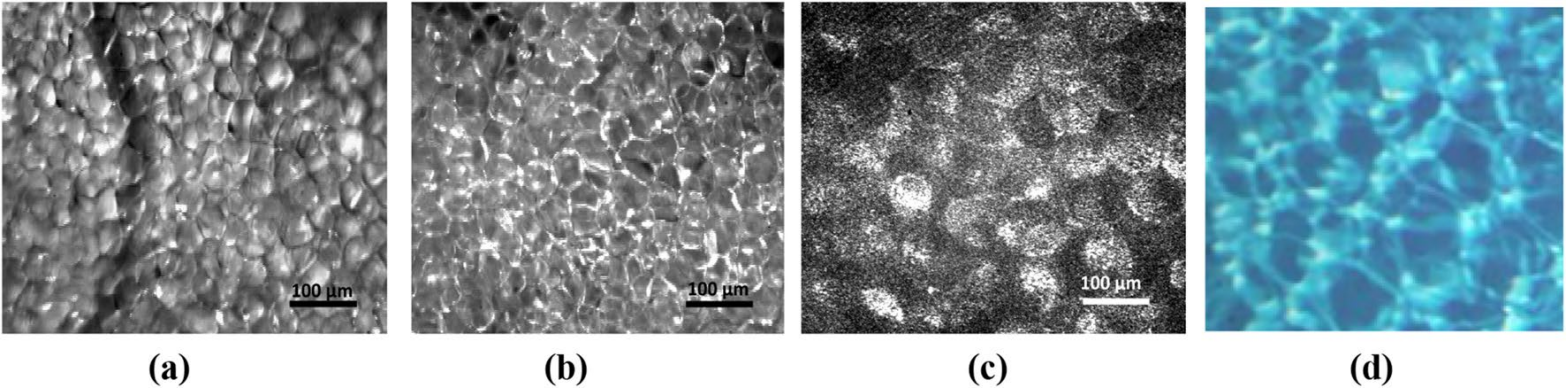
In vivo microscopic images of fat tissue of a rat at back reflectance from a few millimeters of an intact abdominal fat layer using a CMOS camera: a white light source (**a**); green LED 517 nm (**b**); He–Ne laser 632.8 nm (**c**); the light breeze on the shallow seawater on a sunny day (**d**) (see Supporting Information); see the similarity of caustics formation due to focusing of shining light by a lens let array of these complex systems with shape variation in a semiregular manner, due to which phase is different from the out-of-water wavefront.

The AT demonstrates a very complicated spatial structure of reflected or transmitted light^17,18,35,36^. This is caused by the complex character of light propagation from conventional light sources, lasers, light-emitting diodes (LEDs), superluminescent diodes, or swept laser sources with high spatial and low temporal coherence used in optical coherence tomography (OCT) (Fig. 1). Therefore, the development of the approaches for describing light propagation in a complex cellular medium is urgently needed^3,37,38^.

The goal of this study is to demonstrate the complex optical properties of AT at physiological temperatures and in the course of further temperature increases and to design a multicell model describing AT striking optics.

### Fat tissue model for numerical simulation

AT is formed by the aggregation of fat cells (adipocytes), usually in the form of a single droplet of triglycerides. Approximately 95% of an adipocyte cell volume is stored fat (lipids). At human body temperature, adipocyte LDs are a low scattering homogeneous liquid^39^. For humans, the fat cell size is in the range of 15–250 μm; the cell lipid content is in an interval of 0.3–1.24 μg^40^.

**Figure 2:**
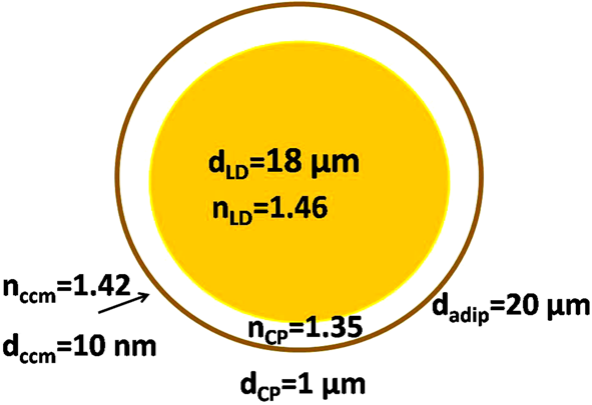
The adipocyte as an optical model: an optical model of adipocyte. CCM is the cell cytoplasmic membrane; CP is the cytoplasm; LD is the lipid droplet; adip is the adipocyte.

Morphologically, WAT adipocytes contain a single large LD, which occupies most of the cell, peripheral cytoplasm, and nucleus, leading to a typical signet ring structure (Fig. 2). LD is a specific organelle consisting of a lipid ester core and a surface phospholipid monolayer; it is not inert storage of excess lipids but is dynamically involved in several cellular functions^41^. Excessive lipid accumulation in LDs is a leading element in the pathogenesis of common metabolic diseases such as obesity, diabetes mellitus, and atherosclerosis^42^.

The literature data for the refractive index *n* of adipocytes and their components are summarized in the supporting information (section S2)^3,35,39^. An LD becomes homogeneous during heating at physiological temperatures, and its refractive index drops. Cool conditions break up AT into individual crystals; the refractive index is higher than the mean.

Adipose cells are generally spherical, with a small bump on one side (Fig. 2)^40^. However, when many cells are together, they press against each other and become polygonal-shaped. In obesity, the volume of adipocytes reaches 300 μm^3^ (vs. approximately 90 μm^3^ in norm)^40^. A single adipocyte with a liquid LD inside looks like an optical microresonator, which confines light within a small cavity. Various adipocyte optical functions*,* including lasing and biochemical/optomechanical sensing, were established in vitro^43^. Therefore, AT is a heterogeneous quasi-regular structure that can be considered a set of lenses combined in columns that can capture light and hold it inside the tissue layer as a lens waveguide^44,45^.

Therefore, it follows the necessity to have adequate models of the interaction of optical radiation with AT to understand its optical properties. Various models of light interaction with complex structures of spherical^46^ and nonspherical^47^ particle arrays can be found in the literature. These photonic models are prospective for describing more comprehensive cell conglomerate optics.

### The forward propagation of light

Since the structure of AT obviously implies a more or less ordered arrangement of cells, it should be expected that the ordering of the elements of the tissue structure will have a significant effect on the overall distribution of light in it. Numerical simulation of analogous dielectric structures in many cases assumes a rigid ordering of structural elements (Fig. 4a), while in real cellular structures the degree of ordering may not be so strong. Nevertheless, it is necessary to understand how taking into account the orderliness of fat cells will affect the results of numerical simulation of light propagation in AT using models close to reality.

**Figure 3:**
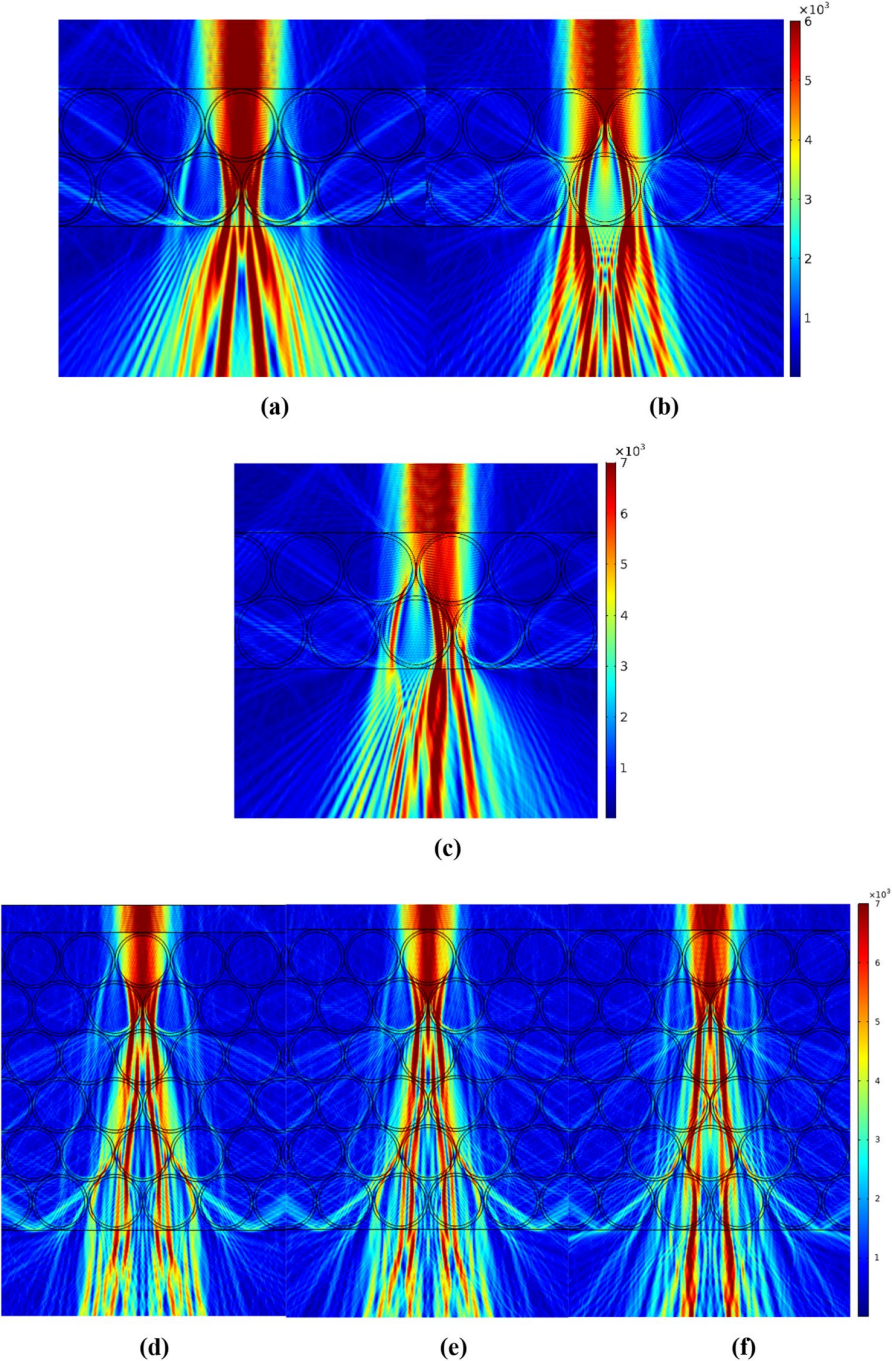
Simulations of Gaussian light beam propagation (waist of the beam is of 10 μm) through 2D fat tissue model (3-layer cell model at immersion in ISF with *n* = 1.36): two cell layer tissue model (**a**)-(**c**) and six-cell layer tissue model (**d**); symmetric [(**a**), (**b**) and (**d**)], and nonsymmetric light beam incidence (c); incidence to cell center in the upper layer (**a**) and (**d**); incidence between two adjusted cells (**b**); six-cell layer tissue model (**e**) and (**f**) with the incidence to cell center in the upper layer at immersion in ISF with *n* = 1.38 and 1.40, respectively.

In this study, the following AT model was developed. Fat cells were represented as 3-layered dielectric spheres with a total diameter of 20.02 μm (Fig. 3). The first outer layer was 10 nm in thickness with a refractive index of *n* = 1.42 (cell cytoplasmic membrane), the middle layer was 1 μm in thickness with *n* = 1.35 (cytoplasm itself),and the inner core was 18 μm in diameter with *n* = 1.46 (model of LD). All the cell layers were considered to be immersed in a medium with a refractive index *n* = 1.36 (model of interstitial fluid (ISF)). In numerical modeling, a tissue model was represented as 2–6 layers of the spheres mentioned above (10 spheres in each layer), considered closely packed. To illustrate the effects of optical clearing caused by the leakage of lipids from cells into the interstitial space when the tissue is heated, the refractive index of the ISF was changed up to *n* = 1.40.

The finite element method (FEM)^48^ was used to solve Maxwell's equations to make a numerical model. The model description is given in the Materials and Methods section.

### OCT imaging model of fat tissue layers for numerical simulation

The description of the numerical simulation approach for the OCT study of AT is presented in supporting information Fig. S-2. Monte Carlo trials for OCT A-scan simulation are conducted. The OCT source incident light beam diameter was 12 μm at the tissue surface and 10 μm in the focus area placed at 100 μm-depth.

## Results

### Numerical simulation for the forward propagation of light

The system of modeled cells was irradiated with a monochromatic continuous wave Gaussian beam with a waist of 10 μm and wavelength 930 nm. These parameters are characteristic of the OCT system used in the experimental study.

For simplicity of the numerical calculations and aiming to obtain a qualitative estimation of light transfer through the system, the 2D case was considered. As shown in Fig. 3, light can propagate sidewaysbymultiple reflections and coupling into whispering gallery modes. Gaussian beam propagation through a layer of large (in comparison with the wavelength) scatterers with a refractive index contrast < 2 enables jet formation^7^. Their shape and direction strongly depend on the initial beam position^47^, and there are three possible scenarios:

1. Direct hitting a cell (Fig. 3a). In this case, the cell focuses the beam into a jet, which, in turn, becomes scattered by the next two cells delivering three bright spots on the bottom surface of the sample (see Fig. 3a). Increasing the number of layers (Fig. 3d) drives beam splitting into a larger number of bright areas symmetrically placed over the interface. Noteworthy that the hot spots take place both inside and outside the cells.
2. Hitting the intercellular volume (Fig. 3b). Under this scenario, the beam is defocused due to interaction with two adjacent curved boundaries and propagates along the cell surfaces. In this case, bright spots occur between the cells emulating the waveguiding effect of the intercellular volume (having a lower refractive index than the cells). However, the full-field picture (Fig. 3b) reveals the true nature of the effect resulting from jet-like beam splitting.
3. Mixed scenario (Fig. 3c). In this case, a part of the beam goes through a cell and a part – outside it, leading to a nonsymmetrical distribution of the bright spots over the bottom interface. Moreover, this type of propagation is supposed to dominate in experimental studies due to the inhomogeneous distribution of cells and their size dispersion in real tissues.

Therefore, in all three cases, we observe beam broadening and splitting into a series of narrow jets giving rise to bright (hot) spots distributed over cells and intercellular areas. The presented calculations qualitatively explain the experimental results in terms of the formation of hot spots upon reflection from the rear boundary of the cell layer and the change in the total transmission of the light beam with a change in the refractive index of the ISF when integrating the output photonic jets over an area equal to the width (20 μm) of the undisturbed irradiating light beam at the bottom of the cell layer and approximately equal to the cross-section of a single cell (see Fig. 3d–f and Table 1).

**Table 1:** The collimated transmission of the light beam with a change in the refractive index mismatch between ISF and LD when integrating the output photonic jets over an area equal to the width (20 μm) of the undisturbed irradiating light beam at the bottom of the cell layer [see Fig. 3d–f] with a total thickness of 120 μm.

Refractive index of ISF, *n*	1.36	1.38	1.40
*n*_LD_/*n*_ISF_ (*n*_LD_ = 1.46)	1.073	1.058	1.042
Transmittance *T* (%) from numerical modelling (Fig. 3d–f)	46.9	48.7	60.3
Transmittance *T* (%) estimated from Eqs. (1) and (2)	52.3	66.8	80.2

The results presented in Table 1 illustrate well that the matching of the RIs of LDs and the environment in the form of an ISF (*n*_LD_/*n*_ISF_) determines not only the nature of the formation of photonic jets as it is shown in Fig. 3, but also the integral collimated transmittance *T*, which follows from numerical simulation for a layer of cells with a thickness *l* = 120 μm. The same table shows the data following from a simple estimate of the transmittance of a layer of particles from the Bouguer-Beer Lambert law for the same thickness of a layer of particles with the same RI ratios (*n*_LD_/*n*_ISF_) under the assumption that there is no absorption and light beam is attenuated mostly due to scattering, and the density and sizes of the scatterers do not change:^3^

1 $$ T = I\left( l \right)/I_{0} \cong \exp \left( { - \mu_{s} l} \right), $$

where *I*_0_ and *I* (*l*) are the intensities of the incident and transmitted light beams and $\mu_{s}$ is the tissue scattering coefficient, which can be estimated for Mie scattering particles as:^3^

2 $$ \mu_{s} \cong a^{2} \rho_{s} \left( {\frac{{n_{LD} }}{{n_{ISF} }} - 1} \right)^{2} , $$

where *a* is the particle radius and $\rho_{s}$ is the particle density; *n*_LD_ is the RI of the LD of AT cells presenting scattering particles and *n*_ISF_ is the RI of interstitial fluid, where these particles are immersed.

It is well seen from Table 1 that a very rough estimation is well fit to numerical modelling, and for both calculations RI matching causes better integral optical transmittance.

### Numerical simulation of OCT-imaging

In this case, adipose cells were modeled by inhomogeneous spheres with three soft-boundary components describing a LD in the center, a thin layer of cytoplasm around it, and a cell membrane in combination with protein intercellular septa as the outer layer of the cell – external shall of the cell (see Supporting Information, Sect. 4.5).

**Figure 4:**
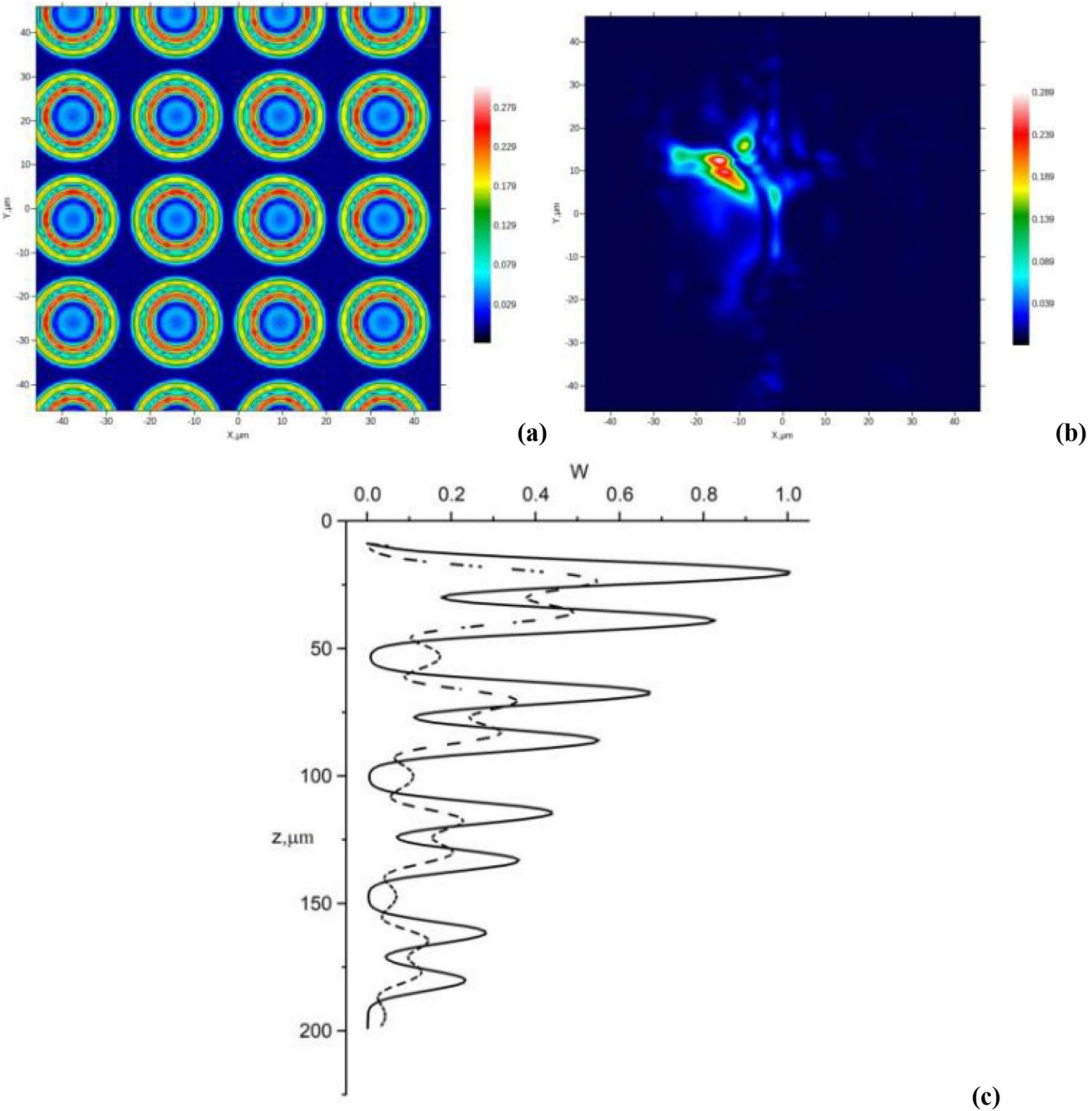
Numerical simulation of OCT-images of AT layers: the reflection coefficient $R(100\mu m,{\mathbf{r}}_{ \bot } )$ at the 100 μm-depth (**a**), and the transverse structure of the reflected from this layer light beam (**b**) for AT fragment with 8 cell-layers along *z*-direction; examples of the A-scans for AT(**c**); the OCT light beam position on the upper surface of AT corresponds to the cell center (dash line) or between two cells (solid line) for AT fragment with 8 cell-layers along the *z*-direction.

The reflection coefficient of the tissue layer $R(100\mu m,{\mathbf{r}}_{ \bot } )$ and the transversal structure of the reflected light beam are shown in Fig. 4. Obviously, the light beamis reflected mainly from the cells' edges.

The A-scan described above for the AT fragment is presented in Fig. 4c. The OCT beam position on the upper surface of the AT corresponds to the cell center (dashed line) or is placed between two cells (solid line). Here, $W = Log_{10} (1 + W_{oct} ({\mathbf{r}}_{ \bot } ,z)/W_{oct} ({\mathbf{r}}_{ \bot } ,0))$ is the OCT interference signal ($W_{oct} ({\mathbf{r}}_{ \bot } ,L)$ is the informative part of the interference signal between the reference and sample optical waves).

The paired peaks on the dashed curve correspond to reflection from the upper and bottom cell boundaries. On the other hand, only single peaks are seen on the solid curve due to the reflection on cells' side borders.

**Figure 5:**
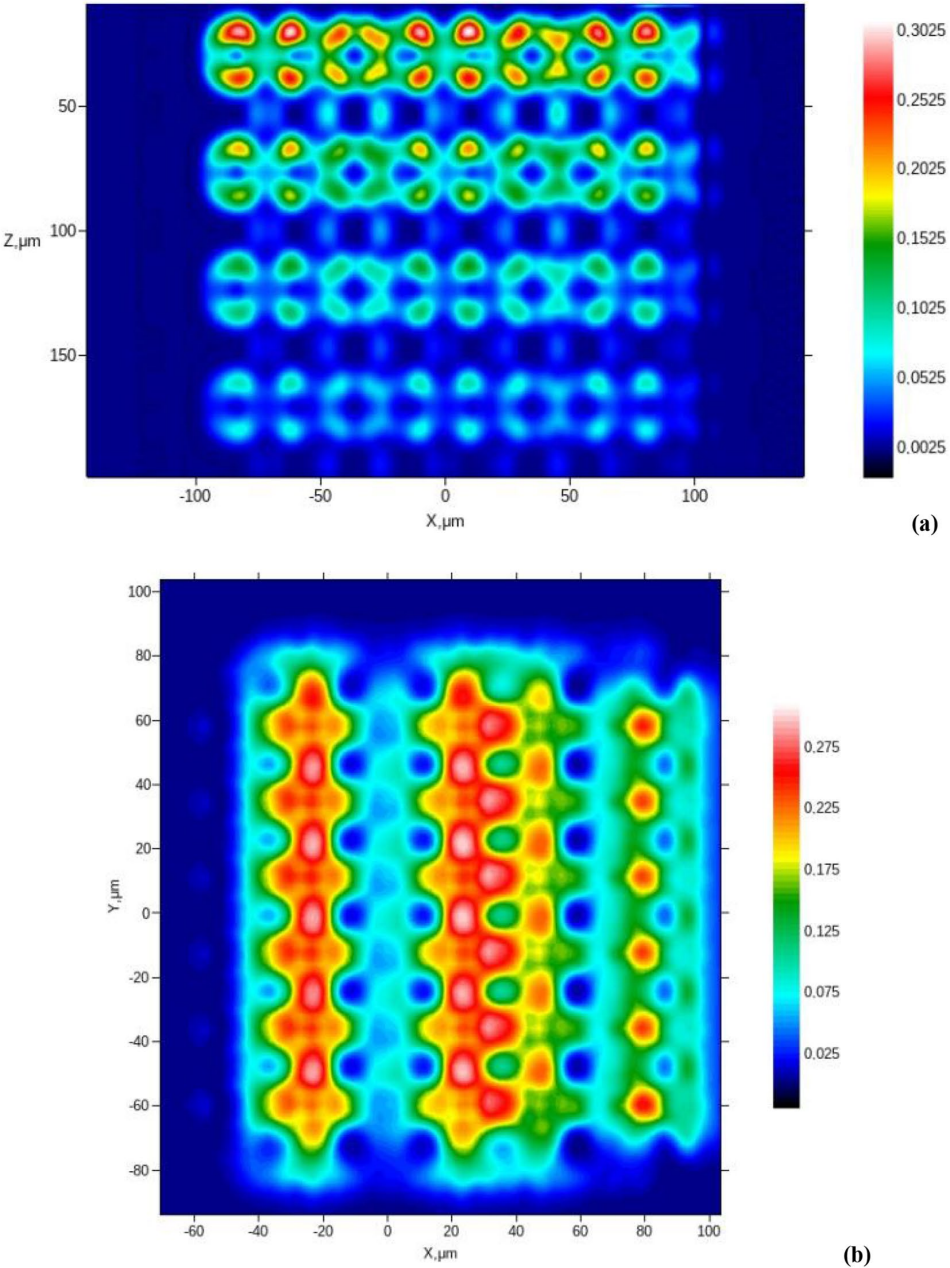
Results of numerical simulation of OCT-images of AT layers: (**a**) example of the B-scan for AT fragment with 8 cell-layers along the *z-*direction; (**b**) the C-scan (in terms of $W$ values) of the 100 μm-depth layer for AT fragment with 8 cell-layers along the *z*-direction.

A B-scan (in terms of $W$ values) of the AT fragment described above is presented in Fig. 5a. A C-scan (in terms of $W$ values) of the 100 μm-deep layer is shown in Fig. 5b. This image corresponds to 441 positions of the OCT source beam on the AT fragment surface.

### An experimental OCT study of ex vivo AT samples

Lipids of cell droplets are characterized by relatively low melting points, which can significantly affect the heating kinetics of tissue with fat accumulation. When heated from 24 to 45 °C, the lipid component of AT undergoes several phase transitions^49^. A series of OCT measurements were performed to study the phase transitions of AT using a commercially available spectral OCT system with a central wavelength of 930 ± 5 nm, the axial resolution of 6.2 μm in air, and transverse resolution of 9 μm. As the mean refractive index of AT in this wavelength range is *n* = 1.47^35^, the actual axial resolution can be estimated as (6.2 μm)/*n* = 4.2 μm.

**Figure 6:**
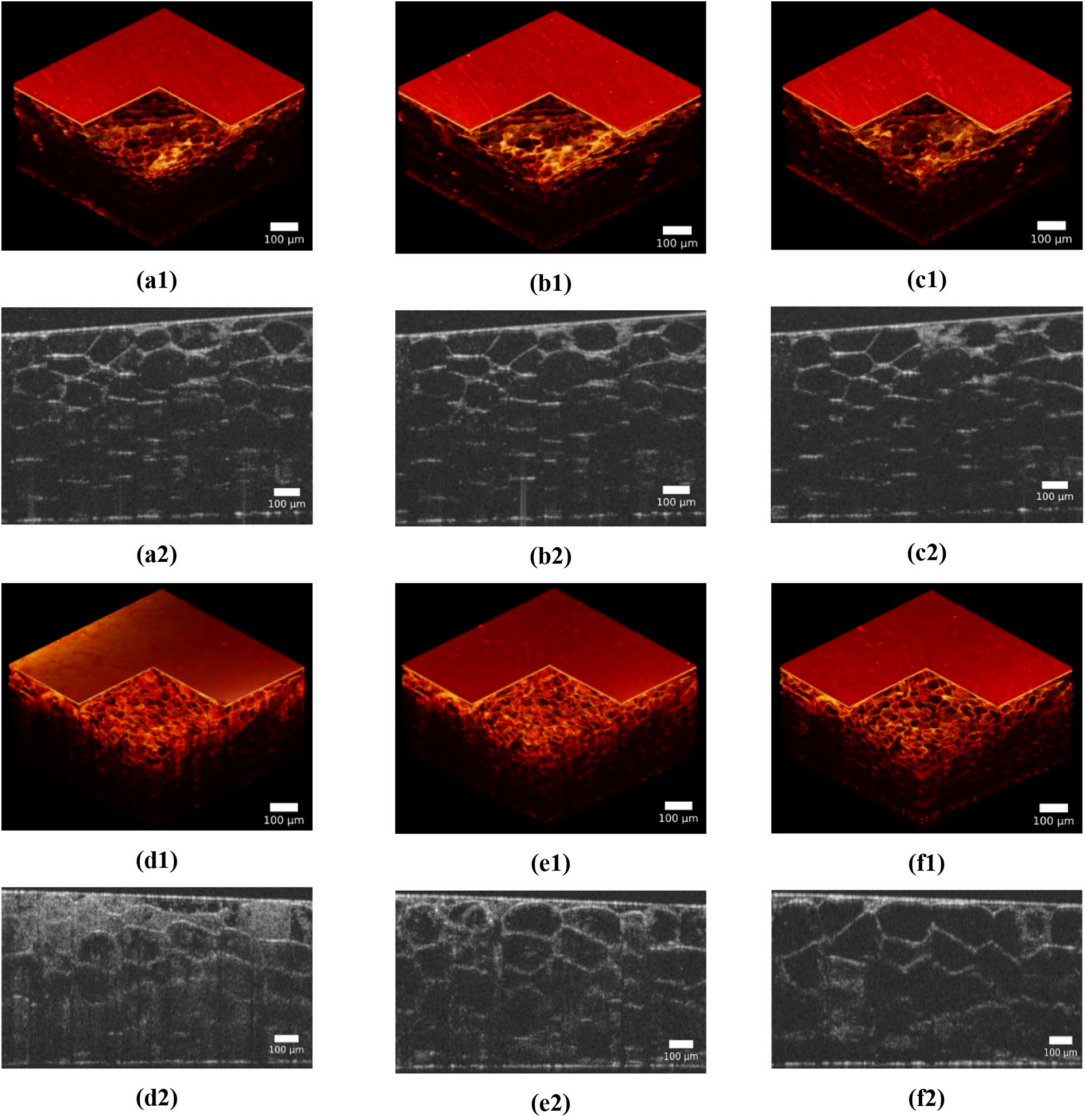
Ex vivo OCT images of human (**a**–**c**) and porcine (**d**–**f**) fat tissue slices for different temperatures: 23 °C (**a**); 30 °C (**b**); 38 °C (**c**); 25 °C (**d**); 30 °C (**e**); 38 °C (**f**). (**a1**)–(**f1**) typical 3D images; (**a2**)–(**f2**) typical B-scans.

**Figure 7:**
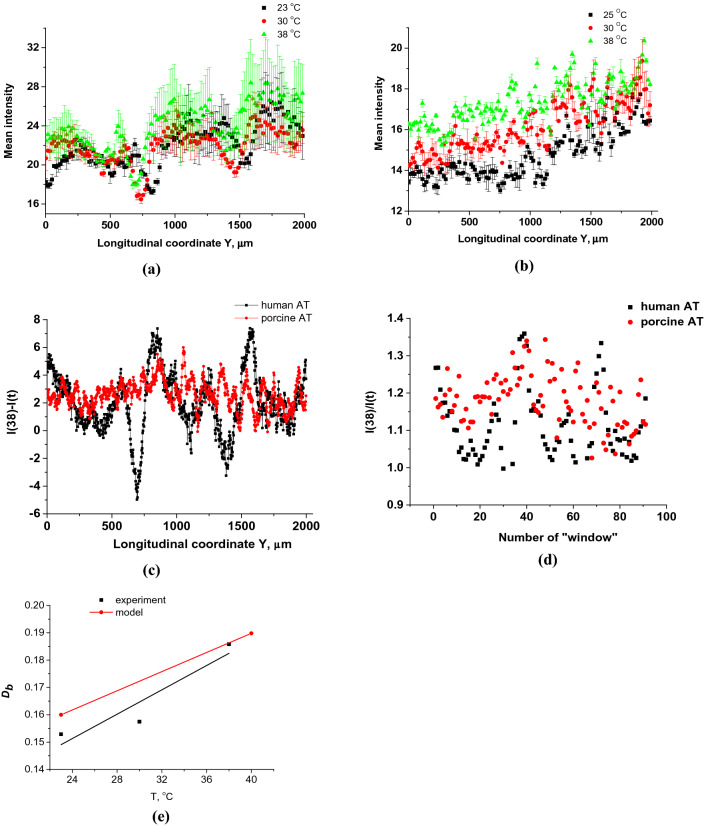
Results of statistical analysis for intensity of all light spots at the bottom of OCT image of the AT samples (see Fig. 7, the bottom surface of the sample image); the evolution with the temperature of these light spots intensity distributions for human (**a**) and porcine (**b**) fat tissue slices is shown. To recalculate the optical thickness into a geometric one, we took the average refractive index of AT equal to 1.44. (**c**) Distribution of difference in intensity at 38 °C and room temperature *t *of light spots at the bottom of OCT image of the AT samples, where* t *= 23 °C for human AT, and 25°C for porcine AT (**a**, **b**, the bottom surface of the sample image). (**d**) Light spot intensity ratio *I*(38)/*I*(*t*) distribution on the number of the averaging window of 20 μm in width counted from 0 to 2000 μm of the longitudinal coordinate of (**a**, **b**), where *t* = 23 °C for human AT, and 25 °C for porcine AT (see Fig. 7, the bottom surface of the sample image), and S-8b and S-8c. (**e**) The results of the ${D}_{b}$ calculation for AT model shown in Fig. S-4b and for experimental data presented in (**a**).

Figure 6 shows a set of 3D OCT images and B-scans of human and porcine fat with a temperature increase (from room temperature to 40 °C). At the bottom surface of the samples, bright light spots show the intensity of localized light sub-beams transmitted through the sample reflected on the bottom interface. The increase in the light spot intensity and their displacement in space occurs when the sample is heated. Averaging was performed over a group of 4 B-scans acquired at the same spatial location of the sample to reduce noise artifacts. A custom histogram equalization algorithm was applied to enhance the quality of the reconstructed images. The top left corner of the B-scan (50 × 50 px) with no sample inside was used as a noise reference. The mean intensity value inside this region was used as a lower intensity boundary. The upper boundary was set to the maximum intensity along the rest of the image pixels. The histogram of the initial B-scan was linearly stretched between these two boundaries to enhance the contrast. The temperature dependence of the light spot intensity is shown in Fig. 7. A monotonic increase in the average brightness value by 20% and 21% is observed for porcine and human tissue samples, respectively. In contrast to human AT, a standard deviation increase is observed for porcine fat samples with temperature elevation.

To quantify variations of quasi-random brightness of the AT OCT B-scans, we used the averaged relative dispersion of an B***-***scan image:

3 $$ D_{b} = \frac{{\sqrt {\langle (\Delta w_{b} )^{2} \rangle } }}{{\langle w_{b} \rangle }} $$

where *w*_*b*_ is the local brightness of the *B-*scan image.

The results of the *D*_*b*_ calculation for AT model presented in Fig. S-4b and for experimental data given in Fig. 7a are presented in Fig. 7e. There is a qualitative agreement between the calculations and experimental data, in which, with increasing temperature, the magnitude of fluctuations in the intensity of the B-scan image increases significantly.

### In vivo microscopy of AT

**Figure 8:**
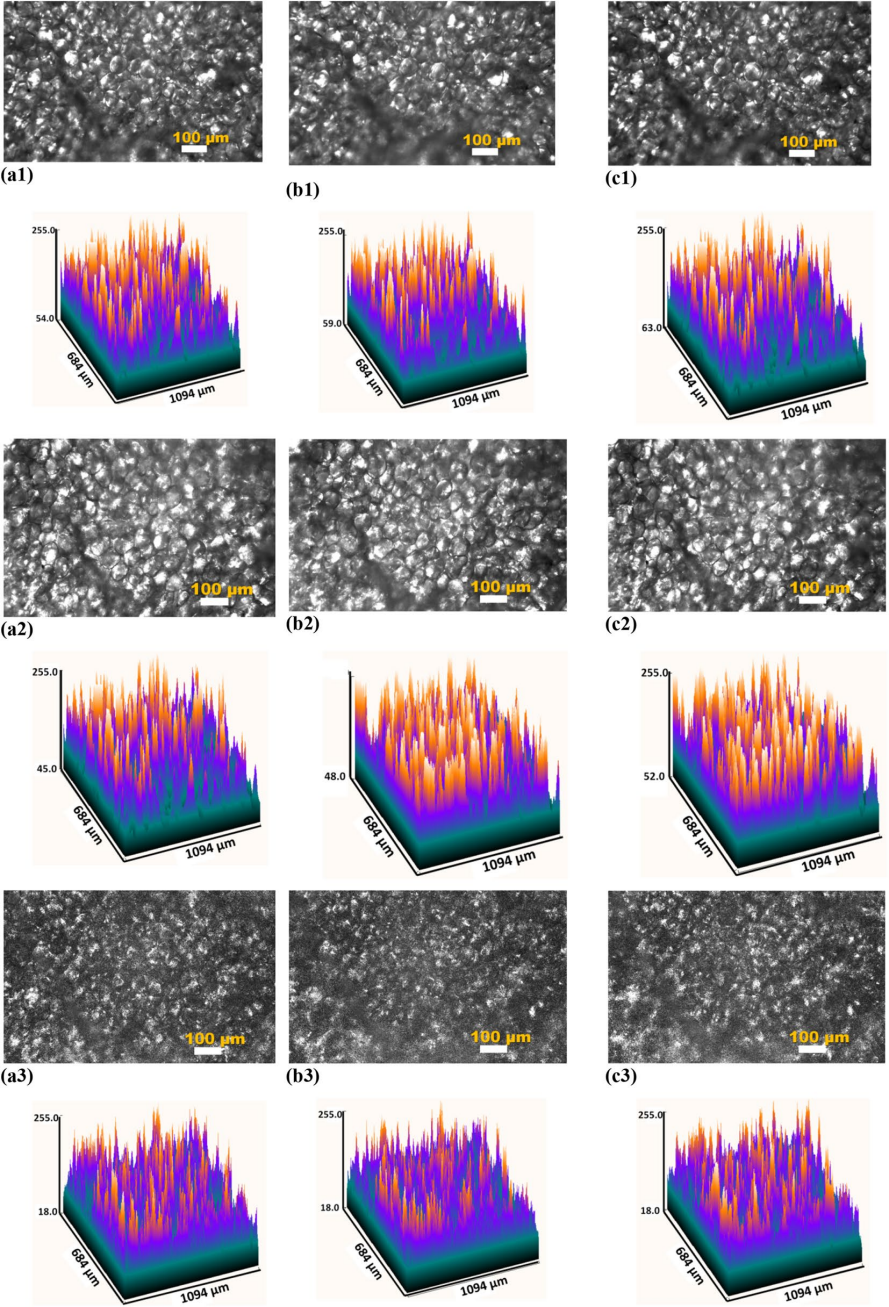

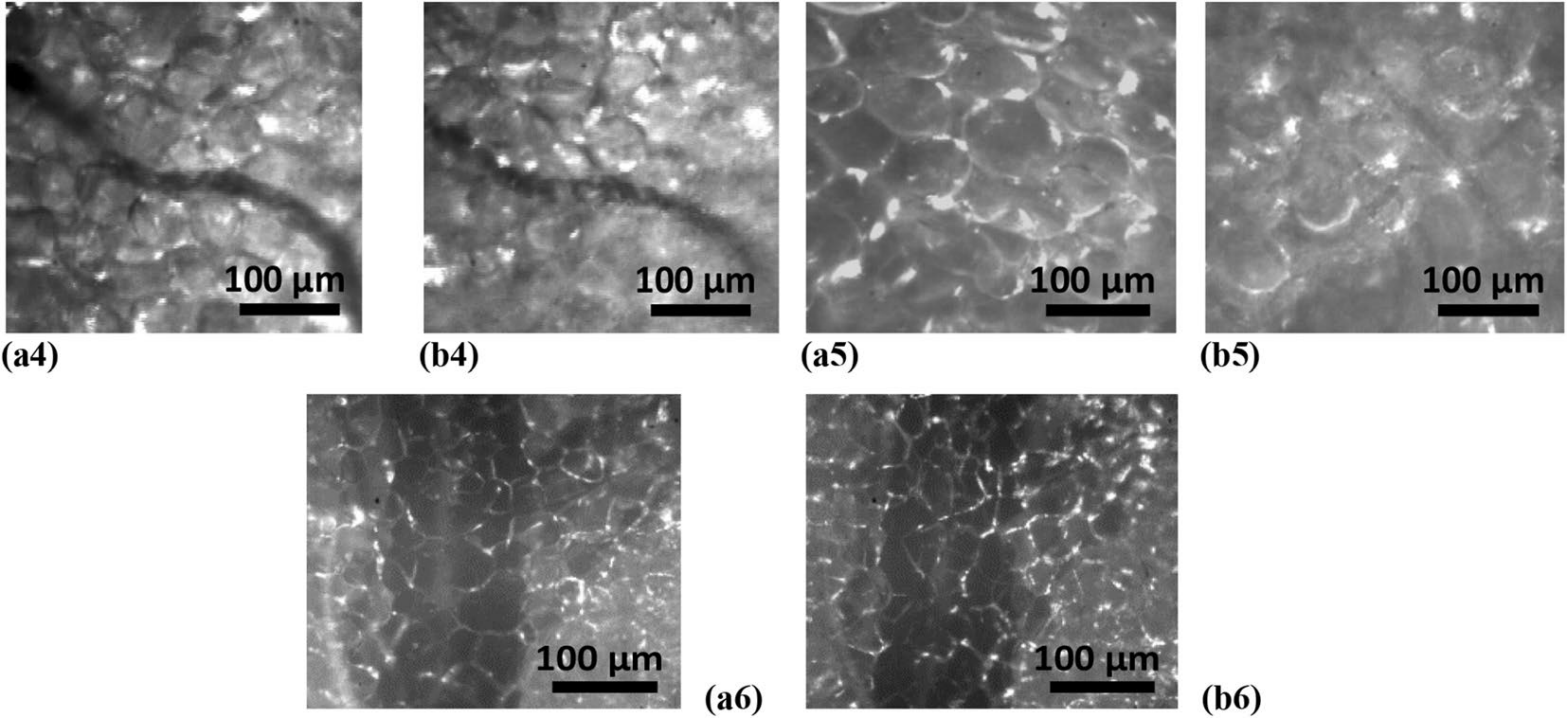
In vivo transmission images of rat abdominal AT sites (cell layer thickness is of 120 ± 15 μm) in the initial state at room temperature (25 °C) (**a**) and after heating up to 30 °C (**b**) and 38 °C (**c**). These images were obtained using the CS235MU monochrome CMOS camera KiraluxTM, the number of pixels in the matrix of 1280 × 1024; 10 bits/pixel (Thorlabs Inc., Newton, New Jersey) with custom software ThorCam 5.6 (Thorlabs Inc., Newton, New Jersey), microscopic objective (lens 10 ×), and white light (**a1**, **b1**, **c1**), green LED with the wavelength of 517 nm(**a2**, **b2**, **c2**), light of He–Ne laser (632.8 nm) (**a3**, **b3**, **c3**). The corresponding 2D distributions of image brigtness were built using ImageJ. *In vivo* back reflectance images of rat abdominal AT (cell layer thickness is of 100 ± 15 μm) in the initial state (**a4**) and after hot saline solution (50 °C) application (**b4**); the initial state (**a5**) and after compression with a fiber tip (**b5**); image of another rat abdominal AT in the initial state (**a6**) and after applying the immersion optical clearing agent PEG-300 (**b6**) (the corresponding video are presented in Supporting Information). These images were obtained using the Basler A602f. monochrome CMOS camera (the number of pixels in the matrix of 656 × 491; 8 bits/pixel) with custom software made with National Instruments LabVIEW 8.5, microscopic objective (lens 10 × ), and green LED with the wavelength of 517 nm.

*In *In vivo transmission images of rat abdominal AT for the cell layer thickness of 120 ± 15 μm, in the initial state at room temperature (25 °C) (a) and after heating up to 30 °C (b) and 38 °C (c), are presented in Fig. 8.

When heated, the transmission of a layer of AT for red light increases, while for white and green light it decreases, and for green it is not as strong as for white. Red light from a laser is weakly absorbed by living tissue, since its wavelength lies at a sufficient distance from the main absorption bands of hemoglobin, and therefore well demonstrates the effect of immersion optical clearing, due to the matching of the RIs of the LD and ISF. However, when probing with white and narrow-band green light by hemoglobin, the flush of blood during tissue heating leads to a strong absorption of light, which makes it impossible in this case to observe the effect of immersion optical clearing, and the correlation coefficients between transmission and tissue scattering coefficient turn out to be negative. The stronger effect of hemoglobin absorption for white light is due to its absorption by the strongest hemoglobin bands—the Soret band and the Q-bands. For narrow-band green light, the absorption is less, since only the Q-bands of hemoglobin affect its absorption.

Figure 8 also shows in vivo backreflectance images of rat abdominal AT before (Fig. 8(a4)) and after application of hot saline (50 °C) (Fig. 8(b4)), before (Fig. 8(a5)) and after compression (Fig. 8(b5)), before (Fig. 8(a6)) and after application of the PEG-300 immersion optical clearing agent (Fig. 8(b6). All these actions can be used to control transmission of layers of living AT in vivo ^50^.

## Discussion

According to numerical simulation, when light propagates in a forward direction in AT tissue, the whispering gallery effects in AT cells dramatically change the characteristics of the propagating light. It could be noted that the shape and position in the space of the photonic jet depend on the illumination conditions. It is visible in Fig. 3 that when the width of the irradiating beam is less than the diameter of the sphere, the formed local region of the focused field deviates from the optical axis (direction of incidence of radiation) and bends in space. Thus, under real conditions, the structure of the field between the layers of cells is complex, caused not only by interference effects but also by the peculiarities of the formation of a localized field by an array of particles (cells). It also partially explains that the local focus areas are demultiplexed in subsequent layers with normal light beam incidence on the first array of spheres.Thus, it can be seen that the optical properties of fat cell layers are rather complex, and rigorousnumerical modeling is required to describe them.

Results of modeling the propagation of light intissue at cell immersion in ISF with different refractive indices *n* = 1.36, 1.38 and 1.40 are shown in Fig. 3d–f. This is a model of tissue optical clearing due to lipolysis of adipocytes when they are heated. Cell lipolysis stimulates the release of a part of the intracellular fluid, including droplet lipid content, into the intercellular space, and the tissue is gradually subjected to immersion optical clearing. As the refractive index of the ISF approaches that of the fat cell, the cell structurebecomes more optically homogeneous and, as a result, more transparent^51^. The matching of RIs of LD and ISF (relative RI *n*_LD_/*n*_ISF_) determines not only the transformation of photonic jets, but also the integral collimated transmittance *T* (Table 1). This has been well proven for the in vivo experimental studies shown in Fig. 3 and in Table 2. When heated, the transmission of the AT layer for red light increases due to the matching of the RIs of the LD and ISF. However, this phenomenon during AT heating could be caused not only by lipolysis, but also by the temperature dependence of RI of the LD^35^.

**Table 2:** The transmittance *T* (%) of the laser beam (632.8 nm) through a rat AT layer of thickness 120 ± 15 μm) (in vivo study) with tissue heating (see Fig. 8).

Temperature, °C	25	30	38
*T*, %	39.4	40.1	41.4
RI of the lipid droplets, *n*_LD_ (taken from Ref ^35^.)	1.467	1.464	1.461
n_LD_/n_ISF_ (reconstracted using Eqs. (1) and (2) for measured *T* values)	1.088	1.087	1.086

Based on the data presented in Fig. 7a,b, the following conclusions can be drawn about the relationship between the model (see Table 1) and experimental data. When samples were heated from room temperature to 38 °C, an increase in the mean intensity, averaged within the scanning line (0–2 mm) with a sampling window of 20 μm, equal to the width of the undisturbed light beamat the bottom cell layer, was found for human AT as (1.11 ± 0.09)-fold, and for porcine AT as (1.18 ± 0.07)-fold (see Fig. 7c,d). These data are in agreement with the ratio of transmitted intensities equal to 1.29, which follows from the calculations when the initial refractive index of the ISF changes from *n* = 1.36 to *n* = 1.40. Approximately the same increase in intensity is also observed for hot spots. It should be noted that the position of hot spots shifts in space when the tissue is heated, which is associated with a change in the conditions of light interference with a decrease in the refractive index mismatch of a spatially inhomogeneous medium. For human AT, a strong redistribution of photonic jets is observed, since the cells behave like good lenses. For porcine AT, cells are not very good lenses, they are largely cloudy due to the higher percentage of fatty acids with a higher melting point (see Table S-1). A tendency for an increase in the mean transmitted intensity with matching of refractive indices has been proven, however, the quantitative differences in this increase and measurement errors between human and porcine ATs are associated with their different melting temperatures since a higher melting temperature causes greater light scattering, which leads to spatial averaging of photonic jets and an elevation of transmittance increase with a simultaneous decrease in the error, which is observed for porcine AT [(1.18 ± 0.07)-fold].

The numerical simulation of OCT AT tissue visualization combines:

Forward propagation of light describes optical beam transformation due to various interaction effects with AT tissue, including whispering gallery effects and photonic jets formation.

- Reflection on the shell of AT cells.
- Backward propagation, which looks similar to forward one.
- Interference with reference light beam.

Therefore, the two numerical models presented above are closely related. In the result of the OCT simulation, we see quasi-regular light spots correlated with the spatial positions of AT cells. Therefore, OCT tools with acceptable characteristics have the potential to measure in vivo AT cells' dimension and spatial distribution. This phenomenon can give a way to detect cell-level pathological processes at an early stage. Examples are apoptosis and cell damage by a virus invasion. In addition, the specific features of the formation of OCT images of cellular structures can serve to monitor the destruction of AT under the action of encapsulated lipase^52^.

The optical clearing of AT demonstrated above during its physiological heating or as a result of the action of specific optical clearing agents, or local compression^3^, can be used for optical monitoring of drug delivery. The capsules may contain both an optical clearing agent and a vaccine.

We used a model of the equidistant spatial distribution of cells of the same size, which, strictly speaking, does not fully correspond to the real situation. Along with the regular model, we used a model of cells with irregular shape, diameter and position by a random cell layer compression and coordinates (x,y) variation. The scale of the random deformation is ~ 20%. The latter means that a scale of random spatial shifts is about 5 μm. We did not established an essential difference between the regular and quasi-regular models. The principal result is that the brightness of the OCT image is strictly inhomogeneous due to the combining effects of photonic jets in one cell and the overlap of these photon jets reflected from refractive index "steps" in the cell structure. This is exactly the case when whispering gallery effects should be taken into account when light propagates in AT cells. Experimental results (Figs. 6, 7) confirm that OCT images of AT tissue have a non-uniform brightness distribution with quasi-regular hot spots. In addition, we see that the environment surrounding cells has a great influence not only on the redistribution of photonic jets but also on the average transmittance (reflectance) (Table 1).

The experimental OCT application results connected with the temperature dependencies can be explained by the fact that the fats within the AT are a complex mixture of triglycerides. Therefore, a single melting point cannot be observed. The melting temperatures of different ATs are presented in Supplement (Table S1). According to these data, porcine fat undergoes phase transitions in the range of temperatures, 36–45 °C. Phospholipids allocated to the cell membrane may also have input in the overall AT phase transition in the range of 38–42 °C. The difference in the thermal behavior of humans and porcine AT is associated with different triglyceride compositions. An increased proportion of unsaturated fatty acid residues in triglycerides reduces the melting temperature of human AT^53^.

The low-temperature transitions in the range of 25–35 °C are associated with fusible free fat acids (FFAs), such as oleic acid (see Table S1 in Supplement).

For the moderate temperature of approximately 40 °C, the phase transitions are defined by the cell membrane phospholipids, whereas for the high-temperature range (45–55 °C), the phase transitions are identified by less fusible FFAs of the fat droplet, such as palmitic acid.

We anticipate that a higher temperature phase transition of 35 °C for human abdominal fat is most likely associated with a cell membrane structure containing phospholipids. Therefore, experimentally discovered multiple phase transitions in AT at tissue heating are attributed to lipids in the cell lipid droplets and phospholipids in the cell membranes. The magnitude of the fluctuations of the B-scan image was shown in experiment and numerical simulation to be increased significantly with temperature increase.

It follows from the analysis of the literature that in many cases the optical parameters of AT were measured at room temperatures^3,15^. At the same time, surgical operations, including various liposuction technologies, are carried out at physiological or even higher than physiological temperatures, at which phase transitions occur in lipids. The present study was conducted at physiological temperatures, so the data obtained can be used for in vivo procedures in the clinic. To model the optical parameters of AT at physiological temperatures, experimental data on the refractive index of a lipid droplet obtained over a wide range of temperatures and wavelengths will be useful^35^.

The results of conventional microscopy of AT in vivo demonstrate the same bright light hotspots as in the OCT images. These hotspots are also associated with the shells of AT cells. We also established that optical microscopy has the ability to control the optics of AT with local heating, compression, or the action of a hyperosmotic optical clearing agent. When the tissue is heated, the image of the vessels lying behind the fat cells is improved, and some details appear; when the AT layer is pressed with the tip of the fiber, the vessels previously hidden behind the cell layer become visible; and the effect of the optical clearing agent increases the contrast of the image of a large vessel. All of these methods can be used in laser surgery or any other surgery with endoscopic maintenance to avoid damage to large vessels during tissue incisions. It is well known that all vital organs are surrounded by fatty tissue, so it is important to "see" through the fatty tissue and be careful when approaching the organ to be operated on.

Recently, Bykov et al. presented an experimentally observed and theoretically confirmed new type of spatial localization of light within biological tissues in vitro^54^. General description of the observed phenomenon based on Monte Carlo ray tracing model was provided. It was found that arrangements of individual adipocytes can act as a cascade of quasi-ordered microscale lenses confining propagation of light within ATs similar to lens lightguides.

Compared to this paper, we performed a rigorous solution to the problem of light transmission through a layer of biological tissue and in vitro and in vivo measurements for human and rat AT (OCT and microscopy), which are in good agreement with model calculations (FEM, Monte Carlo simulations).

## Conclusion

Thus, it can be concluded that AT demonstrates complex optics, which significantly differs from the widely used diffusion optics of tissues^3^. An adequate model of light propagation in quasi-ordered conglomerates of adipose cells can be built based on a multilayer lens grating with waveguide properties. Changing the temperature of the AT, compression and the use of optical clearing agents makes it possible to effectively control the behavior of light propagation in the AT. Such control is crucial both for providing laser light impact on pathological tissues/organs lying behind the layer of fat cells and for obtaining objective information about the results of laser action for building smart laser surgical systems with feedback, including robotic laser systems.

It is important to note that the developed model of cellular tissue can be used not only to describe AT but also to describe epithelial cell layers of internal organs, such as the cervix.

In addition, the developed model can be used to describe the propagation of light in porous tissues, such as the lungs and the brain^55^. The internal structure of the lung is divided into a large number of small subunits (alveoli), the average size of which in humans is commensurate with the size of a fat cell and is approximately 200 microns in diameter. Unlike fat cells, alveoli are filled with air, which creates severe problems for optical imaging, including OCT, due to the high contrast of the refractive index between the walls of the alveoli and the closed area filled with air. For the brain, the pore size of the extracellular medium is slightly smaller than the size of adipocytes and is approximately 1–4 µm, and the refractive index contrast is not so high. In both cases, the refractive index inside the structure is lower than outside, which entails new complex optical effects experimentally observed for soap bubbles under laser excitation.

The presented results can be applied in a new and attractive approach tolabel-freesensing in biology and medicine,medical diagnostics using fat cell biolasing effects^56^, and description of light beams transportation in cellular tissues.

## Methods

### OCT

Abdominal AT samples taken from humans in the course of plastic surgery (5 women, 40–50 years old, 80–90 kg) were used for the OCT study. The experimental protocol was approved by the Ethics Committee of Saratov Medical University (Protocols No. 1 of 03.09.2013, No. 8 of 17.05.2016, No. 1 of 13.09.2016, No. 8 of 10.04.2018). Informed consent was obtained from all subjects and/or their legal guardian(s).All procedures with animals were performed in accordance with "Rules for Conducting Qualitative Clinical Trials in the Russian Federation" (approved by the Ministry of Health of the Russian Federation and enacted on January 1, 1999), the provisions of the WMA Declaration of Helsinki (2000) and the recommendations contained in the European Community Directives (No. 86/609EC). The study is reported in accordance with ARRIVE guidelines (https://arriveguidelines.org).

Before measurements, the fat tissue was stored in a refrigerator at − 28 °C for 3 days. While frozen, the sample slices of fat tissue were cut and slowly warmed to room temperature. The thickness of tissue slices thus prepared was measured using a mechanical micrometer and ranged from 450 to 1050 µm. The number of samples was 10.

For the ex vivo measurements, aSpectral Radar OCT System OCP930SR 022 (Thorlabs Inc., Newton, New Jersey) was used. In this system, the light source is a low-coherence broadband superluminescent diode with a central wavelength (930 ± 5) nm and a spectral bandwidth of 100 nm. The coherence length that determines the axial resolution of the system is 6.2 μm, and the scanning depth is 1.6 mm, both in air. The transverse resolution of the OCT system measured with the help of a microscopic scale was 9 μm, and the output power was 2 mW. As a result of OCT imaging, one obtains a two-dimensional array of the digitized OCT signal with rows corresponding to lateral and axial scanning.

A tissue slice under study was placed into the temperature-stabilizing homemade system (heated specimen holder with glass windows connected to the source of current). The temperature of a sample ranging from room temperature to 40 °C was provided by the corresponding change in the voltage of heating thermistors from 5 to 10 V. The data obtained were processed using MathLab.

### Microscopy

Microscopic studies were carried out both in reflected light and in passing through the AT. In vivo studies were carried out for seven white male rats weighing 180–220 g. In the course of observation, rats were under general anesthesia (Zoletil). During anesthesia, a laboratory animal under study was fixed on a special table; then, the abdominal fat was carefully retrieved by laparotomy onto a stainless steel surface for microscopy back reflectance. Images and videos were obtained using an experimental setup (see Supplement document), including a Basler A602f. monochrome CMOS camera (the number of pixels in the matrix of 656 × 491; 8 bits/pixel) with custom software made with National Instruments LabVIEW 8.5, a microscopic objective (lens 10x, NA = 0.25), and various light sources. A white light source (illumination angle of the investigated surface was approximately 45°), LED with a wavelength of 517 nm (illumination angle of the investigated surface was approximately 90°), and He–Ne laser with a wavelength of 632.8 nm (illumination angle of the investigated surface was approximately 45°) were used. Videos (see Supporting information) and images of abdominal fat tissue from laboratory rats were recorded at a rate of 25 frames per second. A hot saline solution (50 °C) was used for fast tissue heating. The tip of the fiber with a 200 µm diameter was used for living tissue compression. As an optical clearing immersion agent, PEG-300 was used. PEG-300 is a transparent, viscous, colorless liquid that exhibits strong hygroscopic properties that decrease with increasing molecular weight^3,15^. The osmotic pressure of PEG-300 is 52.8 MPa.

In the case of transmission microscopy, the AT was laid out on a special transparent glass table. Strict control of the temperature of AT was carried out using an IR camera (FLIR A300, Sweden). We dripped physical solution temperature heated to 40–45 °C. And then we recorded the image for several seconds. We received several frames at a fixed temperature. The experimental setup (see Fig. S-9(b)) consisted of CS235MU monochrome CMOS camera KiraluxTM with the number of pixels in the matrix of 1280 × 1024; 10 bits/pixel (Thorlabs Inc., Newton, New Jersey) with software ThorCam 5.6 (Thorlabs Inc., Newton, New Jersey) and various light sources: white light source, LED with a wavelength of 517 nm, and He–Ne laser with a wavelength of 632.8 nm. In this case, laser radiation of He–Ne laser and white light were directed to an optical mirror, which redirected the illumination to AT, when using a light source with a wavelength of 517 nm, the LED were placed directly under the transparent surface of the table.

### Modeling

1. To simulate the light distribution for the AT models, shown in Fig. 4 and supporting information Fig. S-1, the finite element method was used^41^. We implemented the 2D scattered field formalism: the Gaussian beam was specified in all calculation domains as if there was free space only. Then, the modeled system of cells (infinite multilayered cylinders) was accounted for as a number of scatterers. This method is widely used in the literature and allows one to investigate the background and scattered (e.g., reflected) fields separately. The domain was surrounded by perfectly matched layers (PMLs) to avoid nonphysical reflections. The finite size of the system and absence of periodic boundary conditions allowed us to simulate a single Gaussian beam without mathematical replications. The commercial software Comsol Multiphysics® was used for the calculations.
2. An adipose cell was modeled by an inhomogeneous sphere with three soft-boundary components describing a lipid droplet in the center, a thin layer of cytoplasm around it, and a cell membrane in combination with protein intercellular septa as the outer layer of the cell. The “soft” model means that the boundaries among cell layers are blurred. A time-domain OCT scheme based on a Michelson interferometer was used to model OCT images of AT cell layers. OCT tissue imaging was simulated using a wave Monte Carlo approach^57,58^. The forward propagation of a sample beam in a Monte Carlo trial was simulated using the “unidirectional Helmholtz equation” approximation^59^. The latter numerical implementation was conducted using the “physical factors splitting” approach^58,59^. The optical reflection coefficient was defined by a medium refractive index gradient. The backward propagation of the sample beam was performed in the same manner. Then, averaging over an ensemble of Monte Carlo trials was fulfilled, and interference with a reference beam was calculated.

## Supplementary Information

Supplementary Information 1.

Supplementary Video 1.

Supplementary Video 2.

Supplementary Video 3.

Supplementary Video 4.

Supplementary Video 5.

Supplementary Video 6.

Supplementary Video 7.

Supplementary Video 8.

Supplementary Video 9.

Supplementary Video 10.
