# Supplementary material for: Light distribution in fat cell layers at physiological temperatures
Source: Sci Rep. 2023 Jan 19;13:1073. doi: 10.1038/s41598-022-25012-9 (PMC9852459; doi:10.1038/s41598-022-25012-9)
Supplement: Supplementary file 1 — Supplementary Information 1. [file 41598_2022_25012_MOESM1_ESM.pdf]

Light distribution in fat cell layers at physiological temperatures

Irina Yu. Yanina[1,2]*, Polina A. Dyachenko[1,2], Arkady S. Abdurashitov[3], Alexander S. Shalin[4,5,6], Igor V. Minin[7,8], Oleg V. Minin[7,8], Andrey D. Bulygin[2,9], Denis A.Vrazhnov[2,10], Yury V. Kistenev[2,10], Valery V. Tuchin[1,2,11,12]

[1]Science Medical Center, Saratov State University, 410012 Saratov, Russia

[2]Laboratory of Laser Molecular Imaging and Machine Learning, National Research Tomsk State University, 634050 Tomsk, Russia

[3]Center for Neurobiology and Brain Restoration, Skolkovo Institute of Science and Technology, 121205 Moscow, Russia

[4] Center for Photonics and 2D Materials, Moscow Institute of Physics and Technology, Dolgoprudny 141700, Russia

[5]Institute of Telecommunications, Riga Technical University, 12 Azenes str., LV-1658 Riga, Latvia

[6]Laboratory of Fiber Optics and Optical measurements UB-1, Kotel'nikov Institute of Radio Engineering and Electronics of Russian Academy of Sciences (Ulyanovsk branch), 432011 Ulyanovsk, Russia

[7]School of Nondestructive Testing, Tomsk Polytechnic University, 634050 Tomsk, Russia

[8]Institute for Strategic Studies, Siberian State University of Geosystems and Technologies, 630108 Novosibirsk, Russia

[9]Laboratory of Nonlinear Optical Interactions, V.E. Zuev Institute of Atmospheric Optics of Siberian Branch of the Russian Academy of Sciences, 634055 Tomsk, Russia

[10]Laboratory for Remote Sensing of the Environment, V.E. Zuev Institute of Atmospheric Optics of Siberian Branch of the Russian Academy of Sciences, 634055 Tomsk, Russia

[11]Laboratory of Laser Diagnostics of Technical and Living Systems, Institute of Precision Mechanics and Control, FRC "Saratov Scientific Centre of the Russian Academy of Sciences," 410028 Saratov, Russia

[12]A.N. Bach Institute of Biochemistry, FRC "Fundamentals of Biotechnology" of the Russian Academy of Sciences, 119991 Moscow, Russia

*Corresponding author

Email: irina-yanina@list.ru

1. Optics of adipocytes

The following data for ATs refractive index (*n*) are available in literature:(1.453±0.001)[1] (assumed at 589 nm) and 1.455[2] at 633 nm) for bovine fat; (1.492±0.003) (at 633 nm),[3] 1.47 (at 633 nm),[4] (1.493±0.005) (at 633 nm),[5] 1.464 (at 633 nm),[6] and (1.467±0.003) (at 633 nm)[7] for porcine fat; (1.467±0.008) (at 633 nm)[8] and 1.46[9,10] (at 442 and 520 nm) for human fat; and (1.467±0.026)[11] (at 800 nm) for rat fat. On average, the refractive index of AT can be evaluated as 1.468±0.013 for room temperature. In the assumption that the refractive index of interstitial fluid is in the range 1.35-1.36,[12,13] the refractive index of the AT scatterers (lipid crystals at room temperature) can be estimated as 1.485±0.008.[14,15] The refractive indices of the sliced porcine and human AT for the temperature 25°C at the wavelength 589 nm were found to be (1.4726±0.0052) and (1.4677±0.0004), respectively.[16] The refractive index of melted porcine fat (i.e., close to the refractive index of the content of lipid droplets) at 25°C at the same wavelength (1.4707±0.0036) is very close to those of the tissue slice. Melting temperatures of porcine and human fats are presented in Table S-1.

2. Specific micro-optic effects in adipocytes

The periodical electromagnetic field localization near the clusters of spherical dielectric microparticles with optical contrast corresponds to the fat cells or, in particular model case, cylinders are shown in Fig. S-1.

3. Numerical modeling of optical wave forward propagation

3.1. The geometrical optics approximation simulation

In this section, analytic equations for the numerical modeling are developed. Recently, field localization in subwavelength volume (FLS) near the shadow surface of low loss dielectric particle of mesoscale dimensions has attracted a considerable amount of research interest due to the plenty of potential applications, including (but not limited to) biomedical devices, Raman spectroscopy, super-resolution particle microscopy, nanoparticle manipulation, etc. The modern state-of-art and an extensive list of literature describing this effect are given in Ref.[20]. It has also been shown that FLS confinement by spherical or cylindrical particles is a complex scattering and

diffraction phenomenon caused by the constructive interference between the incident, scattered, and diffracted fields near the dielectric particle[21] due to the near-field interactions at the particle's boundary. Furthermore, the specific geometrical shape of the scatterers influences the optical field patterns in the FLS region.[20-22]

On the other hand, in the framework of the geometrical optics approximation (GOA),[20,23,24] for dielectric spheres (3D-body) with the refractive index contrast $1 < n < 2$ illuminated by a light wave, the external focus appears at some distance from particle shadow-side surface. In the case of $n = 2$, the focal point is situated exactly at the shadow rim of the spherical lens in this approximation. Analogously, for $n > 2$ the incident light is focused inside the spherical particle. Applying Snell's law of light refraction (in fact, on the refractive index contrast), it can be shown in the GOA that the focus is situated at a distance $f = \frac{R}{2}\frac{n}{n-1}$ (as measured from the center of the sphere with the radius R).

The optical field enhancement $I_{\max}^{sph}$ at the focal point calculated by the GOA ($n < 2$)[20] equals to:

$$\frac{I_{\max}^{sph}}{I_0} \approx \frac{27n^4}{\left(4-n^2\right)^3}\left\{\frac{1, n<2}{\frac{n^2}{2n\left(n-\sqrt{2}\right)+2}, n>\sqrt{2}}\right. . \quad \text{(S-1)}$$

For greater clarity, the equation (S-1) is written in a parametric form, where refractive index contrast n has a role of parameter. As the focus position is inside the spherical particle, in the case of $n > \sqrt{2}$, we are more interested in another case where $n<2$. In this case, equation (S1) can be written as

$$\frac{I_{\max}^{sph}}{I_0} \approx \frac{27n^4}{\left(4-n^2\right)^3} \quad .\text{(S-2)}$$

Similarly, in the case of an infinite circular cylinder (2D-geometry), a formula for the field enhancement $I_{\max}^{cyl}$ at $n<2$ can be written as

$$\frac{I_{\max}^{cyl}}{I_0} \approx \sqrt{\frac{27n^4}{\left(4-n^2\right)^3}} \quad . \tag{S-3}$$

From these expressions and under the approximations mentioned above, one can see that the maximal field enhancement is realized just near the particle boundary. The magnitude of field enhancement depends only on particle refractive index contrast, while the focal waist is proportional to the particle radius and can be significantly smaller than half of wavelength.[20]

Also Eqs. (S-2) and (S-3) clearly demonstrate the fundamental difference between 2D and 3D geometries of light wave focusing. Thus, the field enhancement for cylindrical particle (2D) is about square of that in a sphere (3D) for the GOA:[25]

$$\frac{I_{\max}^{cyl}}{I_0} = \sqrt{\frac{I_{\max}^{sph}}{I_0}} \quad . \tag{S-4}$$

At the same time, both spherical and cylindrical particles (as cubic and rectangle cylinders) qualitatively possess similar characteristics of a localized field.[25] However, due to the mesoscale size of the dielectric particle-lenses, their focusing behavior cannot be explained within the GOA only.[20,23,25] Indeed, according to the GOA, the field enhancement in the focus does not depend on particle size parameter [see Eqs. (S-1) and (S-2)]. In contrast, the wave theory yields strong dependence of the field enhancement on the size parameter.[20,24]

Thus, the analysis of light localization near the shadow surface of a single and chain of low loss 2D and 3D particles studied in the paperwas based on generally accepted theory today that the interference between the scattered or diffracted wave with the incident wave is the leading cause for the formation of FLS.[20-25]

The transmission of a Gaussian beam of constant radius ($w_0 = w_a$) through a chain of dielectric particles can be considered as an inhomogeneous dielectric medium, where w_a and w_0 are the radii of a Gaussian beam at the location of the particle-lens and in the waist, respectively[26]:

$$w_0(z) = w_a\sqrt{1+\left(\frac{z}{L_R}\right)^2} \quad , \tag{S-5}$$

where L_R is the Rayleigh length.

In the approximation of thin particles, it can be shown that the effective refractive index of such a dielectric medium $n(y)$, consisting of a chain of particles, decreases from the axis (y is the distance to the axis) according to the parabolic law and is equal to that from Ref.[27]:

$$n(y) = 1 + \frac{r^2\lambda^2}{2{w_0}^4\pi^2}\left(1 - \left(\frac{y}{r}\right)^2\right), \tag{S-6}$$

where r is the radius of a particle-lens and λ is the illuminating wavelength. It could be noted that there is a curious situation in the sense that the parabolic law takes a special place among the gradient index (GRIN) fibers, as it leads to the periodic focusing of the waveguide modes.[28]

At the optical axis (y=0), the equation (S-2) predict the required refractive index contrast for this structure via relative radius of the particle (r/w_0) and corresponding relative wavelength (λ/w_0) in the unit of Gaussian beam radius:

$$n(0) = 1 + \frac{r^2\lambda^2}{2{w_0}^4\pi^2}\ . \tag{S-7}$$

For example, from Eq. (S-3) at the condition of $r/w_0 = 2$, it is followed that the maximal value of refractive index contrast in the optical axis is $n(0)$=1.01 ~ 1.03.[29] Full-wave simulations shown that the maximal value of refractive index contrast in the optical axis may be even more – up to $n(0)$~1.1. The effect of periodical focusing modes in the chain of low-loss dielectric cylindrical particles with refractive index contrast near unity is illustrated in Fig. 3 in the main text.

4. OCT numerical simulations

4.1 OCT simulation scheme

A time-domain OCT scheme based on a Michelson interferometer was used for numerical simulation of OCT images of AT cell layers. Here, we used the approach based on the wave Monte Carlo implementation when the OCT A-scan is calculated for a probing light beam with a given initial random phase.[30] The Monte Carlo trials for OCT A-scan simulation for the required initial random phase distribution were

conducted. Then, averaging over all OCT A-scan realizations was produced. The OCT modeling is based on the following steps (Fig.S-2):

1. The definition of the initial parameters for OCT imaging model.
2. The definition of the spatial distribution of absorption coefficient and refractive index of AT cell model.
3. The 3D-back reflectance calculation associated with the used AT cell model.
4. A-scan simulation:

4.1 A reference beam optical path tuning for selection of the initial imaging depth;

4.2 The application of the Monte Carlo trials cycle to simulate a necessary degree of coherence of the light beam:

4.2.1 Random variation of the initial phase front to fit the required degree of spatial and temporal coherence;

4.2.2 Simulation of direct propagation of the sampling light beam based on the unidirectional Helmholtz equation numerical solution and AT cell model;

4.2.3 Simulation of thesecondary light beams reflected by different AT layers, backward propagation based on the numerical solution of the unidirectional Helmholtz equation, and preliminary calculated 3D-reflectivity of tissue;

4.2.4 Calculation of the reference and the secondary light beams interference;

4.3 Averaging over the Monte Carlo trials cycle;

4.4. Repeating steps 4.1-4.3 for the next step of imaging depth.

5. B-scan simulation: the B-scan is composed of a set of A-scans, registered in several points along a line on a tissue surface.
6. C-scan simulation: the C-scan is composed of a set of A-scans for a specific imaging depth, registered in theprojection ofseveral points on a tissue surface.

4.2 An adipose tissue model

An adipose cell was modeled by an inhomogeneous sphere with three soft-boundary components describing a lipid droplet in the center, a thin layer of cytoplasm around it, and a cell membrane in combination with protein intercellular septa as the outer layer of the cell– external shall of the cell. The “soft” model means that the boundaries among cell layers are blurred (Fig.S-3a). When the AT temperature is increased, the refractive index spatial variations in an adipose cell are decreased (Fig.3-4b).

A 9x8x6 identical cell fragment was used in numerical simulation. We used three models of fat tissue:

I) We considered cell shall with an internal diameter near 18 μm and external one – near 22 μm. The odd and even cell layers were shifted horizontally on cell half-size distance. The cells are immersed in a low refractive index interstitial fluid.

II) Along with the regular models, we used a model of cells with irregular shape, diameter and position by a random cell layer compression and coordinates (x,y) variation. The scale of the random deformation is ~20%. The latter means that a scale of random spatial shifts is about 5 μm.

AT refractive index spatial distribution in both models is shown in Figs.S-4, S-5.

4.3 Optical wave beam forward propagation model

Denote ψ - the complex amplitude of the electric field strength of the light beam $E(\boldsymbol{r},t)$:

$$E(\boldsymbol{r},t)=\psi(\boldsymbol{r})e^{-i\omega_0 t}. \tag{S-8}$$

The complex amplitude ψ fulfills the Helmholtz equation

$$\left(\Delta+k_0{}^2\varepsilon\right)\psi=0, \tag{S-9}$$

where $k_0=\omega_0/c$ is the wavenumber, ω_0 is the carrier frequency, c is the speed of light, ε is the medium's complex permittivity. It is convenient to split ε into constant and spatially varied parts: $\varepsilon(\mathbf{r})=\varepsilon_0+\delta\varepsilon(\mathbf{r})$.

Let z-axis be oriented along a direction of light beam propagation. Then Eq. (S-9) can be presented in the form:

$$\partial_z{}^2\psi(\mathrm{z},\mathbf{r}_\perp)=-\left(\Delta_\perp+k_0{}^2\varepsilon_0\right)\psi(\mathrm{z},\mathbf{r}_\perp)-k_0{}^2\delta\varepsilon\psi(\mathrm{z},\mathbf{r}_\perp), \tag{S-10}$$

where $\mathbf{r}_\perp$ is the radius-vector in a plane transverse to the z- axis, $\Delta_\perp=\partial_x{}^2+\partial_y{}^2$ is the transverse Laplacian.

Below, we will take into account only the forward light beam ("unidirectional Helmholtz equation" approximation). This approximation imposes restrictions on the $\psi(z,\boldsymbol{r}_\perp)$ function.

The numerical solution of Eq. (S-10) was based on the physical factors' splitting approach.[31-33] Here, we used two-phase screens: a homogeneous screen which accounts diffraction, absorption, and phase shift associated with ε_o, the second one describes absorption and phase shift associated with $\delta\varepsilon(\mathbf{r})$. The block diagram of the corresponding algorithm is shown in Figure S-6.

The following equation describes the step of light passing an inhomogeneous screen:

$$\partial_z{}^2\psi = -k_0{}^2\delta\varepsilon\psi\,. \tag{S-11}$$

Its solution is:

$$\psi(z+\delta z,\mathbf{r}_\perp) = \psi(z,\mathbf{r}_\perp)\exp(-i\delta z k_0\sqrt{\delta\varepsilon})\,, \tag{S-12}$$

where δz is a numerical greed step along propagation direction of the light beam.

Let define a Fourier transform of $\psi(z,\boldsymbol{r}_\perp)$ function

$$\tilde{\psi}(z,\mathbf{k}_\perp = \iint_\infty \psi(z,\mathbf{r}_\perp)e^{i\mathbf{k}_\perp\mathbf{r}_\perp}d\mathbf{r}_\perp/2\pi\,. \tag{S-13}$$

Then the step of passing a homogeneous screen is described by the equation

$$\partial_z{}^2\tilde{\psi}(z,\mathbf{k}_\perp) = -(\varepsilon_0 k_0{}^2 - \mathbf{k}_\perp{}^2)\tilde{\psi}(z,\mathbf{k}_\perp)\,. \tag{S-14}$$

We need to calculate only a forward wave. In this case, the solution of (S-14)is:

$$\psi(z+\delta z,\mathbf{r}_\perp) = \int_{\mathbf{k}_\perp\in\mathrm{Re}\sqrt{\varepsilon_0 k_0{}^2-\mathbf{k}_\perp{}^2}>0} \mathbf{dk}_\perp e^{-i\mathbf{k}_\perp\mathbf{r}_\perp}\tilde{\psi}(z,\mathbf{k}_\perp)e^{i\delta z\sqrt{\varepsilon_0 k_0{}^2-\mathbf{k}_\perp{}^2}}\,. \tag{S-15}$$

The additional condition $\mathrm{Re}\sqrt{\varepsilon_0 k_0{}^2-\mathbf{k}_\perp{}^2} > 0$ provides the selection of the forward wave.

4.4 Optical wave beam backward propagation model

The optical reflectance of the AT layer $R(z,\mathbf{r}_\perp)$, which connects the complex amplitudes of the forward and backward waves $\psi_R(z,\mathbf{r}_\perp) = R(z,\mathbf{r}_\perp)\psi(z,\mathbf{r}_\perp)$, was calculated:

$$R(z,\mathbf{r}_\perp) = \left| \frac{n(z,\mathbf{r}_\perp) - n(z-\delta z,\mathbf{r}_\perp)}{n(z,\mathbf{r}_\perp) + n(z-\delta z,\mathbf{r}_\perp)} \right|. \qquad \text{(S-16)}$$

This approximation takes into account optical wave components oriented close to z-axes, which are within the detector field of view. Here, $n(\mathrm{z},\mathbf{r}_\perp) = \sqrt{\varepsilon(\mathrm{z},\mathbf{r}_\perp)}$.

The complex amplitude $\psi_R(z,\mathbf{r}_\perp)$ of the backward optical wave was calculated using the same numerical algorithms as for direct wave simulation.

4.5 Interference of the reference and sample light beams

The degree of coherence of the OCT light source can be taken into account by averaging Monte Carlo trials. We also assume that all secondary optical waves reflected from the tissue layer reach the OCT receiver at the same time. Thus, the OCT interference signal:

$$W_{oct}(\mathbf{r}_\perp, L) \approx 2\,\mathrm{Re}\int\limits_{Z} dz_r \int\limits_{\Re} \left\langle \left\{ \hat{G}_\varepsilon^{\Leftarrow} \psi_R^*(\mathbf{r}_\perp, z_r) \right\} \psi_0(\mathbf{r}'_\perp, L) \right\rangle d\mathbf{r}'_\perp \qquad \text{(S-17)}$$

,

where $W_{oct}(\mathbf{r}_\perp, L)$ is the informative part of the interference signal between the reference and sample optical waves; $\hat{G}_\varepsilon^{\Leftarrow}$ is the operator of optical wave transformation during the backward propagation in the medium after the reflection on the layer with z_r coordinate; $\psi_0(\mathbf{r}'_\perp, L)$ is the reference wave complex amplitude, L is the length of the reference arm, symbol "*" means complex conjugation operation, $\langle ... \rangle$ means averaging over an ensemble of Monte Carlo trials.In approximation that all photons reflected from a z_r layer spend the same time to get a detector, the Eq. (S17) can be presented in the form.

$$W_{oct}(\mathbf{r}_\perp, L) \approx 2\,\mathrm{Re}\int\limits_{\infty} \psi_0(\mathbf{r}'_\perp, L)\psi_R^*(\mathbf{r}'_\perp, z-L) g_r(\mathbf{r}_\perp - \mathbf{r}'_\perp) g_t(z-L)\, dz\, d\mathbf{r}'_\perp . \qquad \text{(S-18)}$$

4.6 The results of a fat tissue fragment OCT imaging simulation

The results of a fat tissue fragment OCT imaging simulation are presented in Figures S-7, S-8. We can conclude that OCT images of regular and irregular model have only small differences. Therefore, the regular model is quite acceptable.

5. *In vivo* microscopy of AT

The scheme of an experimental setup for *in vivo* back reflectance and transmission imaging of abdominal fat tissue in rats is present in Figs. S-9.

Figures and figure captions:

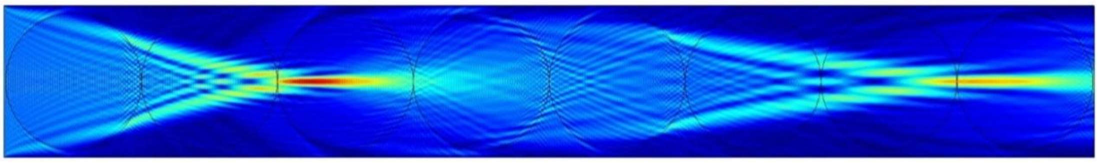

Fig. S-1. Full-wave simulation of periodical focusing mode effect in the chain of dielectric cylinders with refractive index contrast near unity with gradually reducing beam waists and in extremely small propagation losses: illuminating wavelength is 905 nm with Gaussian beam profile, the diameter of cylinders are 18 μm with refractive index 1.46 immersed in water with refractive index 1.33 (refractive index contrast is 1.098). The period of focusing increases with decrease the refractive index contrast.

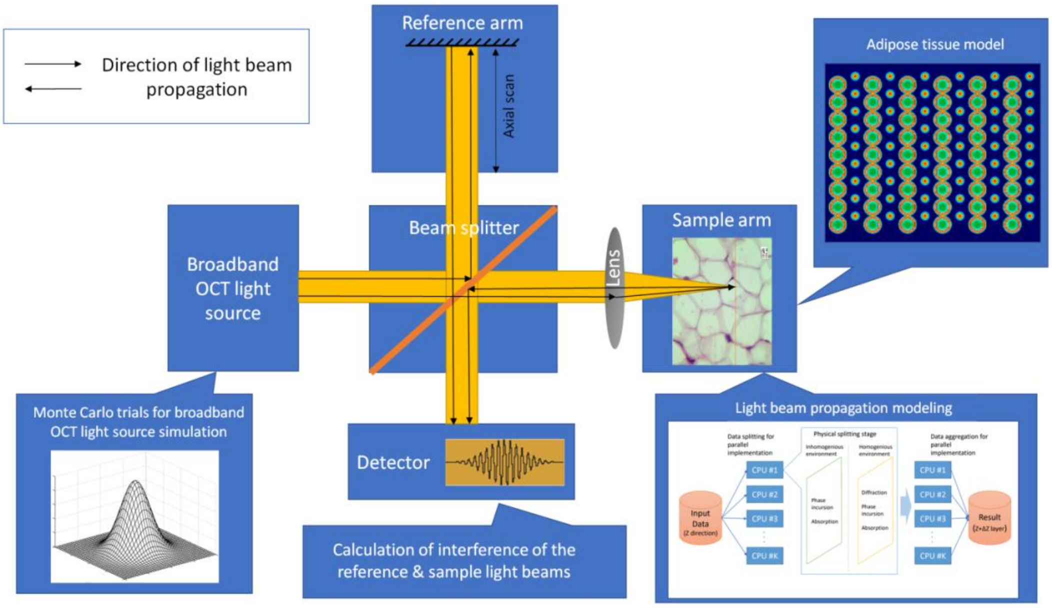

Fig. S-2. A block-scheme for simulation of numerical implementation of OCT A-scan.

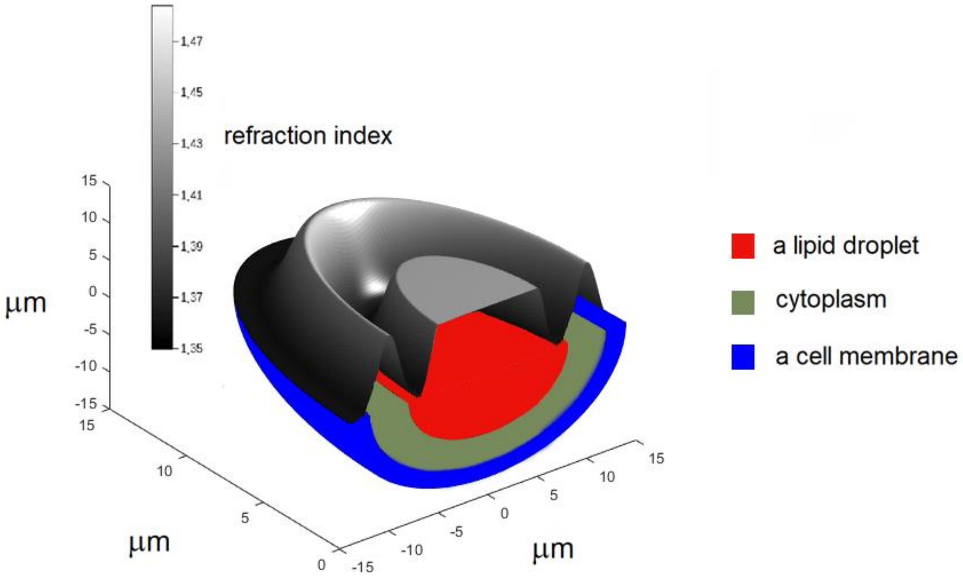

(a)

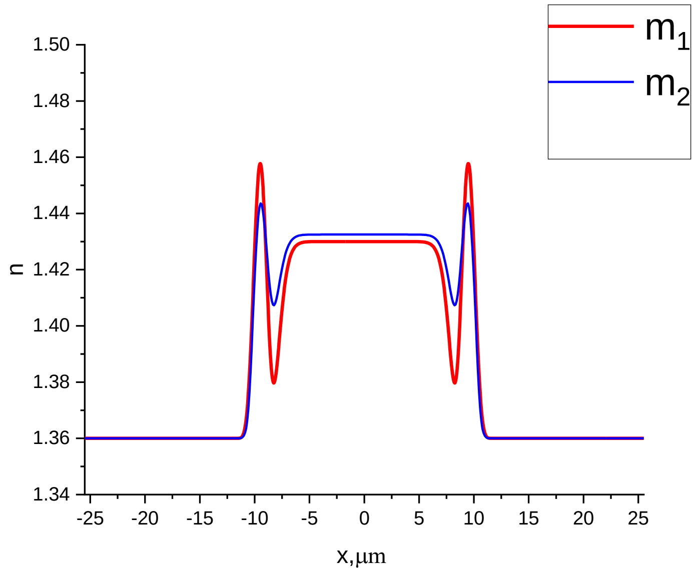

(b)

Fig. S-3. Refraction index spatial distribution of the used “soft boundary" model of a fat cell. (a) The color image describes geometry of the cell; the grey image describes the cell's refraction index. (b) Transformation of a cell’s refraction index spatial distribution, when the cell is heated from a room temperature T_1 to temperatures about 40°C.

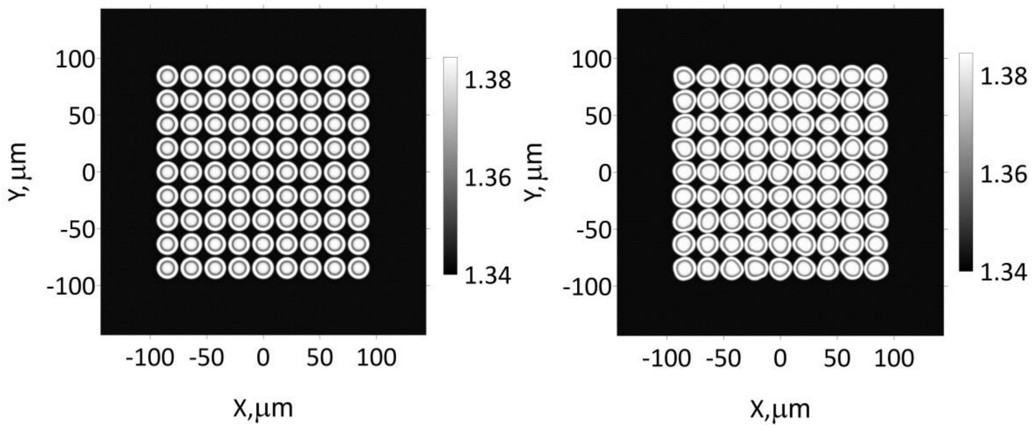

(a)(b)

Fig.S-4. AT refractive index spatial distribution in the plane of z=100 μm at room temperature for: (a) theregular model (I), (b) the quasi-regular model (II).

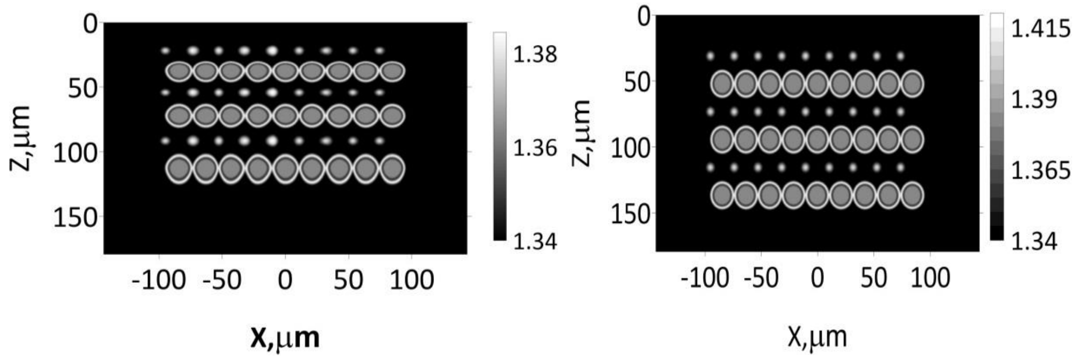

(a) (b)

Fig.S-5. AT refractive index spatial distribution in the plane of x=100 μm at room temperature for: (a)- the regular model (I), (b)- the quasi-regular model (II).

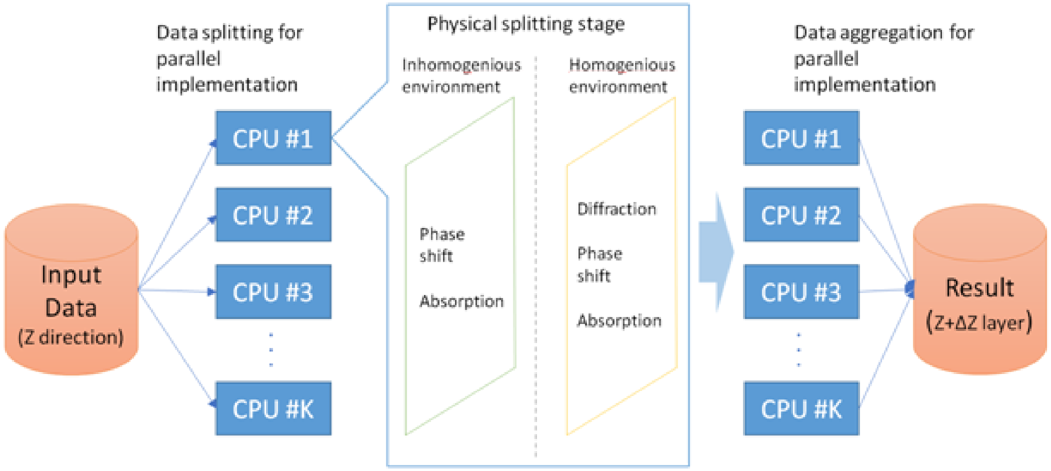

Fig. S-6. The block diagram for physical factors' splitting algorithm.

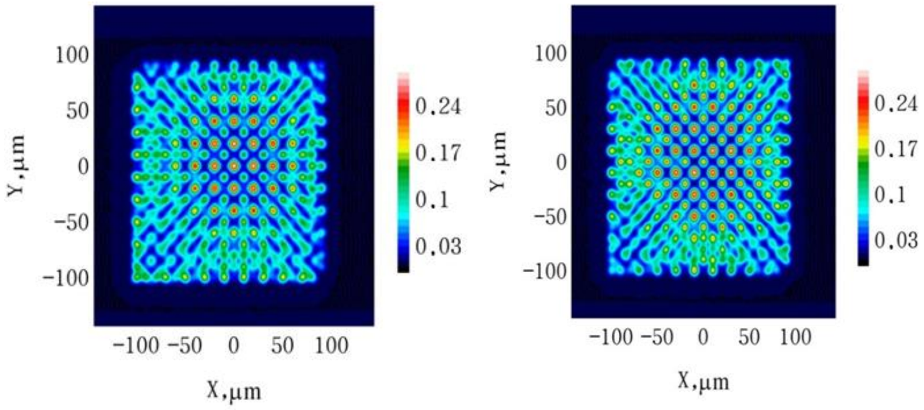

(a) (b)

Fig.S-7. The example of the C-scan on the 100 μm-depthof AT fragment at a room temperature. a)- the regular model (I), b)- the quasi-regular model (II).

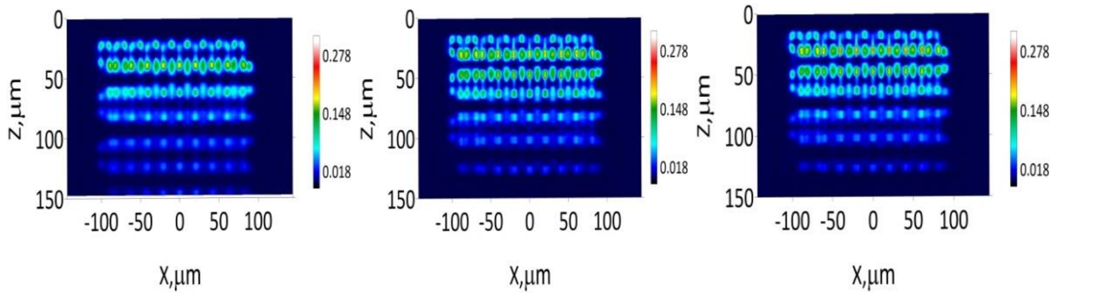

(a) (b) (c)

Fig.S-8. The example of the B-scan of AT fragment at a room temperature: a)- the regular model (I) for room temperature, b)- the quasi-regular model (II) for room temperature; b)- the quasi-regular model (II) for the temperature about 40°C (the model m_1 in Fig. S-3b).

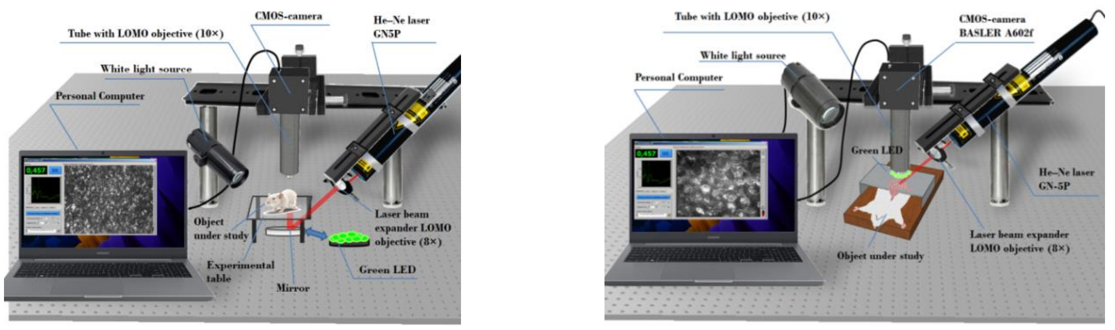

(a) (b)

Fig. S-9. The scheme of an experimental setup for *in vivo* transmission (a) and back reflectance (b) imaging of abdominal fat tissue in rats.

Table. S-1. Melting temperatures of porcine and human fats.[17-19]

Triglycerides	Melting temperature, °C	Free fatty acid (FFA), % by mass (melting temperature, °C)				
		Palmitic	*Stearic*	*Oleic*	*Linoleic*	*Linolenic*
Porcine fat (in solid state)	36-45	27 (63)	14 (70)	45 (16)	5 (-5)	5(-11)
Visceral human fat	30-35	25 (63)	8 (70)	46 (16)	10 (-5)	-

Supplementary_video 1, 2: *In vivo* videos of rat abdominal fat tissue before (1) and after applying a hot saline solution (50ºC) (2) in a white light

Supplementary_video 3, 4: *In vivo* videos of rat abdominal fat tissue before (3) and after applying a hot saline solution (50ºC) (4) in green LED ligh with the wavelength of 517 nm.

Supplementary_video 5, 6: *In vivo* videos of rat abdominal fat tissue before (5) and after applying a hot saline solution (50ºC) (6) in light of He-Ne laser (632.8 nm).

Supplementary_video 7, 8: *In vivo* videos of rat abdominal fat tissue before (7) and after slight compression (8) in green LED light with the wavelength of 517 nm.

Supplementary_video 9, 10: *In vivo* videos of rat abdominal fat tissue before (9) and after applying the PEG-300 immersion optical clearing agent (10) in green LED light with the wavelength of 517 nm.

References

[1] Cook, C. F., Bray, R. W., & Weckel, K. G. Variations in the chemical and physical properties of three bovine lipid depots. *J. Anim. Sci.* **24**, 1192-1194 (1965).

[2] Bolin F. P. et al. Refractive index of some mammalian tissues using a fiber optic cladding method. *Appl. Opt.* **28**, 2297-2303 (1989).

[3] Cheng, S., Shen, H. Y., Zhang, G. , Huang, C. H., Huang, X. J. Measurement of the refractive index of biotissue at four laser wavelengths. Proc. SPIE 4916, 172-176 (2002).

[4] Lai, J. et al. Experimental measurement of the refractive index of biological tissues by total internal reflection. *Appl. Opt.* **44**, 1845-1849 (2005).

[5] Li, H., & Xie, S. Measurement method of the refractive index of biotissue by total internal reflection. *Appl. Opt.* **35**, 1793-1795 (1996).

[6] Lin, L., Li, H., & Xie, S. Linear method of determining the refractive index of biotissue. *Proc.* SPIE **3863**, 177-181 (1999).

[7] Ye, Q. et al. Measurement of the complex refractive index of tissue-mimicking phantoms and biotissue by extended differential total reflection method. *J. Biomed. Opt.* **16**, 097001 (2011).
[8] Tearney, G. J. et al. Determination of the refractive index of highly scattering human tissue by optical coherence tomography. *Opt. Lett* **20**, 2258-2260 (1995).
[9] Yanina, I. Yu., Trunina, N. A., & Tuchin, V. V. Optical coherence tomography of adipose tissue at photodynamic / photothermal treatment *in vitro. J. Innov. Opt. Health Sci.* **6**, 1350010 (2013).
[10] Katika, K. M., & Pilon, L. Steady-state directional diffuse reflectance and fluorescence of human skin. *Appl. Opt.* **45**, 4174-4183 (2006).
[11] Zysk, A. M., Chaney, E. J., & Boppart, S. A. Refractive index of carcinogen-induced rat mammary tumours. *Phys. Med. Biol.* **51**, 2165-2177 (2006).
[12] Tuchin, V. V. *Tissue Optics: Light Scattering Methods and Instruments for Medical Diagnostics* (Bellingham: SPIE Press, 2015).
[13] Yanina, I. Yu., Bochko, V.A., Alander, J.T., & Tuchin, V.V. Optical image analysis of fat cells for indocyanine green mediated near-infrared laser treatment. *Laser Phys. Let.* **8**, 684-690 (2011).
[14] Rogers, M.A., Tang, D., Ahmadi, L., Marangoni, A.G. Fat crystal networks in *Food materials science: principles and practice* (New York: Springer, 2008).
[15] Ribeiro, A. P. et al. Crystallization modifiers in lipid systems. *J. Food. Sci. Technol.* **52,** 3925-3946 (2015).
[16] Yanina, I. Yu., Lazareva, E. N., & Tuchin, V. V. Refractive index of adipose tissue and lipid droplet measured in wide spectral and temperature ranges. *Appl. Opt.* **57,** 4839-4848 (2018).
[17] Turk, S. N. & Smith, S. B. Carcass fatty acid mapping. *Meat Sci.* **81**, 658-663 (2009).
[18] Ojha, S., Budge, H., Symonds, M. E. in *Pathobiology of Human Disease. A Dynamic Encyclopedia of Disease Mechanisms, Part II: Organ Systems Pathophysiology* (Elsevier, Academic Press, 2014).
[19] Castro, G. S. F. et al. Association between hepatic cholesterol and oleic acid in the liver of rats treated with partially hydrogenated vegetable oil.*Rev. Nutr. Campinas.* **25**, 45 (2010).
[20] Lukiyanchuk, B. S. et al. Refractive index less than two: photonic nanojets yesterday, today and tomorrow. *Opt Mater Express* **7**, 1820–1847 (2017).

[21] Devilez, A., et al. Spectral analysis of three-dimensional photonic jets. *Opt.Express***16**, 14200–14212 (2008).

[22] Lecler, S., Takakura, Y. & Meyrueis, P. Properties of a three-dimensional photonic jet. *Opt. Lett.***30**, 2641-2643 (2005).

[23] Kwan, A., Dudley, J., & Lantz, E. Who really discovered Snell's law? *Phys. World* **15**, 64 (2002).

[24] Minin, I. V. & Minin, O. V. *Diffractive Optics and Nanophotonics: Resolution below the Diffraction Limit*. (Cham: Springer, 2016).

[25] Geints, Yu. E. et al. Systematic study and comparison of photonic nanojets produced by dielectric microparticles in 2D- and 3D- spatial configurations. *J. Opt.* **20**, 065606 (2018).

[26] Wenzel, R. G. Oscillations of Gaussian beam parameters in periodic lens waveguides. *Opt. Communs*. **43**, 89–92 (1982).

[27] Smolyaninova, V. N., et al. Experimental demonstration of Luneburg waveguides, *Photonics* **2**(2), 440–448 (2015).

[28] Arfken, G. B., Weber, H. J. *Mathematical Methods for Physicists* (New York: Acad. Press, 1995).

[29] Mikaélyan, A. SELFOC dielectric waveguides. *Sov. J. Quantum Electron*. **7**, 266-267 (1977).

[30] Bulygin, A.D. et al. Imitation of optical coherence tomography images by wave Monte Carlo-based approach implemented with the Leontovich-Fock equation, *Opt. Eng*. **59** (6), 061626-1–12 (2020).

[31] Marchuk, G. Some application of splitting-up methods to the solution of mathematical physics problems, *Appl. Math.* **13**(2), 103–132 (1968).

[32] Strang, G. On the construction and comparison of difference schemes, *SIAM J. Numer. Analys*. **5**, 506–517 (1968).

[33] Marchuk, G. I. Splitting and alternating direction methods, *Handbook Numer. Analys*, Eds P. G. Ciarlet, J.-L. Dons. Amsterdam etc.: North-Holland. **1**, 197–462 (1990).
